# Structural and functional characterization of Mpp75Aa1.1, a putative beta-pore forming protein from *Brevibacillus laterosporus* active against the western corn rootworm

**DOI:** 10.1371/journal.pone.0258052

**Published:** 2021-10-11

**Authors:** Jean-Louis Kouadio, Stephen Duff, Michael Aikins, Meiying Zheng, Timothy Rydel, Danqi Chen, Eric Bretsnyder, Chunsheng Xia, Jun Zhang, Jason Milligan, Artem Evdokimov, Jeffrey Nageotte, Yong Yin, William Moar, Kara Giddings, Yoonseong Park, Agoston Jerga, Jeffrey Haas

**Affiliations:** 1 Bayer Crop Science, Chesterfield, Missouri, United States of America; 2 Department of Entomology, Kansas State University, Manhattan, Kansas, United States of America; University of Texas at Dallas, UNITED STATES

## Abstract

The western corn rootworm (WCR), *Diabrotica virgifera virgifera* LeConte, is a major corn pest of significant economic importance in the United States. The continuous need to control this corn maize pest and the development of field-evolved resistance toward all existing transgenic maize (*Zea mays* L.) expressing *Bacillus thuringiensis* (*Bt*) insecticidal proteins against WCR has prompted the development of new insect-protected crops expressing distinct structural classes of insecticidal proteins. In this current study, we describe the crystal structure and functional characterization of Mpp75Aa1.1, which represents the first corn rootworm (CRW) active insecticidal protein member of the ETX_MTX2 sub-family of beta-pore forming proteins (β-PFPs), and provides new and effective protection against WCR feeding. The Mpp75Aa1.1 crystal structure was solved at 1.94 Å resolution. The Mpp75Aa1.1 is processed at its carboxyl-terminus by WCR midgut proteases, forms an oligomer, and specifically interacts with putative membrane-associated binding partners on the midgut apical microvilli to cause cellular tissue damage resulting in insect death. Alanine substitution of the surface-exposed amino acids W206, Y212, and G217 within the Mpp75Aa1.1 putative receptor binding domain I demonstrates that at least these three amino acids are required for WCR activity. The distinctive spatial arrangement of these amino acids suggests that they are part of a receptor binding epitope, which may be unique to Mpp75Aa1.1 and not present in other ETX_MTX2 proteins that do not have WCR activity. Overall, this work establishes that Mpp75Aa1.1 shares a mode of action consistent with traditional WCR-active Bt proteins despite significant structural differences.

## Introduction

The western corn rootworm (WCR), *Diabrotica virgifera virgifera* LeConte (Coleoptera: Chrysomelidae), is a significant maize pest causing an annual economic loss exceeding 1 billion dollars in the United States [1]. Since 2003, transgenic maize expressing a single (e.g., Cry3Bb1) or dual (e.g., Gpp34Ab1/Tpp35Ab1) *Bacillus thuringiensis* (*Bt*) pore-forming proteins with distinct receptor specificity have provided protection against WCR. WCR has a history of adaptation to multiple control tools and strategies including applied chemical insecticides [2], behavioral adaptation to crop rotation [3], and transgenic crop insecticidal proteins [4–6]. The discovery and development of new insecticidal proteins are therefore needed to protect maize against WCR.

Mpp75Aa1.1 (formerly known as Cry75Aa1.1), discovered by mining the genome of *Brevibacillus laterosporus*, provides effective control of WCR larvae when expressed in transgenic maize [7]. Furthermore, Mpp75Aa1.1 exhibits significant activity toward WCR that are resistant to Cry3Bb1 and Gpp34Ab1/Tpp35Ab1 (formerly known as Cry34Ab1/Cry35Ab1) [7], suggesting a distinct receptor specificity in WCR compared to Cry3Bb1 and Gpp34Ab1/Tpp35Ab1. Bioinformatic analysis identified that Mpp75Aa1.1 belongs to the aerolysin-like superfamily of bacterial beta-pore forming proteins (β-PFPs) found across all kingdoms of life [8,9]. More specifically, the Pfam (Protein family) [10] database classification indicates that it is a member of the ETX_MTX2 sub-family of β-PFPs [11,12]. Despite their limited primary amino acid sequence similarity, β-PFPs share a conserved structural core comprised of two pairs of anti-parallel β-strands and a membrane insertion β-hairpin [8]. Typically, following recognition of a specific binding partner(s), the concentration of these β-PFPs increases in the micro-environment of the cell membrane where oligomerization occurs, and the β-hairpin of each monomer undergoes significant conformational changes while adopting an amphipathic β-hairpin to form transmembrane β-barrel pores [13,14]. Along with a common pore-forming hairpin, β-PFPs of the ETX_MTX2 family comprise a structurally diversified receptor recognition domain integral to their mode of action and conferring specificity on various cell membranes [15]. Furthermore, structural and functional studies have delineated the receptor binding domains of cytocidal [16] and insecticidal [17,18] proteins.

The mode of action of insect-specific β-PFPs from the ETX_MTX2 family has been described for Mpp51Aa2.834_16 (formerly known as Cry51Aa2.834_16) [18], Mpp64Ba/Mpp64Ca (formerly known as Cry64Ba/Cry64Ca) [19], and MTX2 [20,21], which are active against hemipteran (Mpp51Aa2.834_16 and Mpp64Ba/Mpp64Ca) and dipteran species (MTX2). Mpp51Aa2.834_16 forms a stable head-to-tail dimer that is processed at its carboxyl-terminus by *Lygus sp*. salivary proteases to liberate monomers that recognize specific receptors on *Lygus sp*. midgut epithelial membranes, leading to insect death [18]. These toxicity steps for Mpp51Aa2.834_16 are consistent with the primary mode of action of 3-domain Cry proteins as discussed in Vachon et al. [22].

In this report, we describe the crystal structure of Mpp75Aa1.1 and outline the principle steps necessary for insecticidal activity on WCR. Mpp75Aa1.1 was found to be susceptible to proteolytic processing, the activated protein formed oligomers, and bound the microvilli resulting with the sloughing off of the apical microvilli layer in the insect gut lumen consistent with previous reports for the mode of action of WCR-active 3 domain Cry proteins. Additionally, we identified specific amino acids with distinct spatial orientation on the surface of the putative receptor binding region of Mpp75Aa1.1, which are important for insecticidal activity and may be used to recognize receptors on the WCR midgut epithelium.

## Materials and methods

### Expression and purification

Mpp75Aa1.1 (pMON320504) and derived variants described below were expressed in the *E*. *coli* Rosetta^™^2 (DE3) strain using an auto-induction system as described by Studier [23]. Briefly, overnight cultures in Luria-Bertani (LB) liquid medium were used to seed ZYP-5052 auto-induction media [23] supplemented with 100 μg/mL kanamycin and 25 μg/mL chloramphenicol. Cell growth and induction were carried out at 37°C for 3 h and subsequently at 20°C for 44 h. Cells were then harvested by centrifugation and resulting pellets were frozen at -80°C until use. For protein purification, cell pellets were lysed for 30 min at 4°C with a 3:1 (vol/vol) mixture of B-PER^™^ (Bacterial protein extraction reagent, Thermo Scientific) and Y-PER^™^ (Yeast protein extraction reagent, Thermo Scientific) supplemented with 25 mM Tris-HCl, pH 8.5, 200 mM NaCl, 0.1 mg/mL lysozyme, 250 units/mL benzonase (ART.sm nuclease expressed from pMON101670), 2.5 mM MgCl_2_, 1 mM phenylmethylsulfonyl fluoride (PMSF), and 10 mM imidazole. Cell lysates were clarified by centrifugation at 20,000 x g at 4°C for 15 min and the supernatant subjected to His-select^™^ nickel resin (Sigma) affinity purification. Nickel resin eluates were loaded onto a Superdex-75 column (Cytiva) equilibrated with 25 mM sodium carbonate, pH 10.5, and 50 mM NaCl for size-exclusion chromatography at a flow rate of 1 mL/min using an AKTA Pure^™^ fast protein chromatography (FPLC) system (Cytiva). Protein fractions were pooled, concentrated, and buffer exchanged into 25 mM sodium carbonate, pH 10.5, and 25 mM NaCl by diafiltration using Amicon^®^ Ultra-15 centrifugal filters (Millipore Sigma). Protein concentrations were determined using absorbance at 280 nm and the proteins were evaluated by intact molecular weight determination using a Q-Tof LC/MS spectrophotometer (Waters) [24].

### Structure determination

Purified Mpp75Aa1.1 (6 mg/mL) was crystallized using vapor diffusion by sitting drops in 96-well plate Corning 3552 (Hampton Research) at 18°C. Crystallization conditions were sought by testing the crystallization behavior of the protein sample versus plates prefilled with 50 μL of the following commercially-available screens: Crystal Screen HT and PegRx HT (Hampton Research) and JCSC+, Wizard12, and Wizard34 (Rigaku Reagents). Crystal trays were set up using a mosquito crystal robot (TTP Labtech). Structure-quality crystals were obtained using the Wizard34 reagent A2 (30% 2-methyl-2,4-pentanediol, 0.1 M sodium acetate buffer pH 4.6, 0.2 M calcium chloride).

The Mpp75Aa1.1 structure was solved by the single anomalous dispersion (SAD) heavy atom phasing method using X-ray intensity data from a crystal that was briefly soaked in samarium acetate. Crystals were cryo-cooled using ~25% ethylene glycol or glycerol prior to plunge-freezing in liquid nitrogen-filled pucks and shipped in advance of data collection to the synchrotron. Suitable X-ray diffraction data for structure determination were collected by remote means at the Southeast Regional Collaborative Access Team (SER-CAT) 22-ID beamline, which is on the Advanced Photon Source (APS) at the Argonne National Laboratory. These data were processed using the HKL2000 package [25]. Numerous crystals were soaked in heavy atom reagents, and X-ray data collected. The SHARP/auto SHARP program (Global Phasing Limited) [26] was used to assess the suitability of derivative crystal data for structure solution phasing, and to solve the structure using the SAD method. Crystallographic refinement was conducted using refmac5 [27], as implemented in the CCP4 package [28]. Map-fitting and model-building were performed using the Coot program [29]. Iterative refinement and model-building were conducted until the structure was of high quality. The structure of Mpp75Aa1.1 was deposited with PDB/RCSB under the code 7ML9.

### Mutagenesis and expression

A T7 expression system expressing wild-type Mpp75Aa1.1 (pMON320504) was used as a template for mutagenesis. A total of 76 solvent-exposed amino acids in the head region of Mpp75Aa1.1 were targeted for alanine substitution. Clones were synthesized using unimolecular and multipart hot fusion [30]. Mutations were confirmed using Sanger and Next Generation Sequencing (Illumina). Protein expression was initiated with glycerol stocks seeded in 1 mL overnight cultures of Brain Heart Fusion glycerol (BHIG) (BD Bacto) media grown at 37°C at 850 rpm. Auto-induction media (5 mL) supplemented with 50 μg/mL kanamycin and 25 μg/mL chloramphenicol was subsequently inoculated at 1:100 overnight cultures, grown for 48 h at 22°C at 550 rpm. Cells were pelleted by centrifugation at 3,200 x *g*, washed and resuspended in ice-cold 50 mM NaCl to make a 2x concentrated *E*. *coli* cell suspension. Suspensions expressing recombinant Mpp75Aa1.1 wild type and derived variants were diluted with 50 mM NaCl to 1x and 0.25x, arrayed into a 96-well block (Qiagen), and frozen at -80°C until used for WCR insecticidal assay.

### Larval diet bioassay and feeding assessment

Non-diapausing WCR eggs (Waterman, IL) were used for all experiments. Artificial diet bioassays were performed using a 96-well plate containing southern corn rootworm (SCR) larval diet (Frontier Scientific F9757). Molten diet was dispensed at 200 μL per well. Protein or washed whole *E*. *coli* samples (20 μL) were overlaid onto the diet surface and allowed to dry. One neonate larva (< 24 h post-hatch) was infested per well and plates were sealed using pre-punched heat sensitive seals. Plates were subsequently incubated in a dark environmental chamber at 27°C, 60% humidity. Larval mortality and stunting relative to control samples were evaluated six days post infestation [31].

### Brush border membrane preparation

A brush border membrane (BBM) preparation method by English *et al* [32] was adapted for whole neonates. Briefly, frozen WCR neonates were homogenized in ice-cold 5 mM Tris-HCl, pH 7.4, 50 mM sucrose supplemented with cytidine 5’-diphosphocholine (Sigma), protease inhibitor cocktail (Sigma), and PMSF, using three 30 s pulses of a polytron PT 2500E (Kinematica, Inc) homogenizer at 15,000 rpm. One volume of 5 mM Tris-HCl, pH 7.4, 50 mM sucrose was added to homogenates, supplemented with 10 mM CaCl_2_, stirred on ice for 30 min and centrifugated at 4,500 x *g* for 30 min. Cleared lysates were passed through a four-layer cheese cloth and further centrifuged at 27,000 x *g* for 30 min. Resulting pellets were re-suspended in half the previous volume, homogenized on ice with a Dounce homogenizer (Sigma), supplemented with 10 mM CaCl_2_, and further centrifuged at 27,000 x *g* for 30 min. BBM pellets were re-suspended in 0.32 M sucrose, aliquoted, flashed frozen in liquid nitrogen, and stored at -80°C. Total protein concentration was determined using the Bradford assay (Bio-Rad), using bovine serum albumin (BSA) as a standard. Enrichment was determined using the enzyme biomarker aminopeptidase-N (APN) and the leucine-p-nitroanilide (Sigma) as substrates [33]. BBM samples with 23 to 36-fold enrichment were used in binding assays.

### WCR gut fluid collection

Gut fluid collection from WCR was as described by Girard *et al*. [34]. Briefly, dissected midguts (usually 10) from third instar larvae reared on non-traited maize seedling roots, were mixed with 50 μL ice-cold 0.15 M NaCl and gently stirred on ice with a Teflon pestle. Midgut tissues and fluid were centrifuged at 10,000 x *g* for 5 min at 4°C. Resulting supernatant (gut fluid) was carefully collected to avoid contamination from the fat layer. Total protein concentration was determined using the Bradford assay and gut fluid was stored at -80°C. Total Cathepsin B protease activity was assessed using the fluorogenic substrate Z-Arg-Arg-7-amino-methylcoumarin-HCl (Sigma).

### *In vitro* proteolytic processing

Purified protein was treated with gut fluid at a ratio of 4:1 protein:gut fluid (w/w) and incubated at 25°C for the indicated length of time, between 10 and 180 min. Reactions were carried out in 30 mM MES pH 6.0, 50 mM NaCl and stopped with protease inhibitor cocktail (Sigma). For protease inhibition, WCR gut fluid was subjected to a 5 min treatment at 25°C with 14 mM 1-trans-Epoxysuccinyl-ʟ-leucylamido(4-guanidino)butane (E-64) [35] prepared in water or with 50 mM PMSF before adding purified Mpp75Aa1.1 to make a final reaction mixture of 30 mM MES pH 6.0, 50 mM NaCl, 0.28 mM E-64 or 1 mM PMSF. A time course reaction was further performed for 10 min, 30 min, 60 min, 90 min, and 120 min. At each time point, 10 μL of the reaction mixture was aliquoted into a clean tube and supplemented with 1 ul of protease inhibitor cocktail (Sigma), and the reaction sample was run onto 4–20% SDS-PAGE (Bio-Rad). To perform trypsin processing of Mpp75Aa1.1, protein in 25 mM carbonate, pH 8–10.5 and 25 mM NaCl, was incubated with L-(tosylamido-2-phenyl) ethyl chloromethyl ketone (TPCK) treated trypsin (Sigma) at a 200:1 ratio protein:trypsin (w/w) and incubated at room temperature for 15 min unless otherwise indicated. Reactions were stopped with 1 mM PMSF for 30 min at room temperature. In-gel digestion and mass spectrometric analyses were adapted from Shevchenko et al [36]. For insect bioassays, trypsin-processed protein was further buffer-exchanged into the indicated bioassay buffer using a ZEBA^™^ desalting column, 7 kDa MWCO (Thermo Scientific), and quantified by absorbance at 280 nm.

### *In vitro* binding to brush border membrane

To perform in-solution competition binding, 1.5 μM fluorescently-labeled, and trypsin-treated disabled insecticidal protein (DIP) variant [18], Mpp75Aa1.1_K125C_N153C, was competed with increasing concentrations (1.5 μM and 23 μM) of unlabeled and trypsin-treated counterpart in the presence of 3 μg of WCR BBM. Reactions were performed in 25 mM phosphate, pH 6.2 and 25 mM NaCl and in triplicate at room temperature for 1 h. Resulting mixtures were pelleted by centrifugation at 20,000 x *g* for 5 min, washed with the reaction buffer and centrifuged as above before loading onto a 4–20% SDS-PAGE. The fluorescein signal from BBM-bound protein was measured and quantified at 492/515 nm (excitation/emission), normalized to the bound-only sample (no competitor) and plotted as a function of competitor concentration.

### Competition bioassay analysis

Mass action *in vivo* competition was performed as previously described [18,37]. Wild-type Mpp75Aa1.1 (7.35 μg/cm^2^) was mixed with increasing concentrations of DIP variant, Mpp75Aa1.1_K125C_N153C, from 1.83 to 58.8 μg/cm^2^, and fed to neonate WCR using the larval diet bioassay described above.

### Thermal stability assay

Thermal stability of purified wild-type Mpp75Aa1.1 and that of the variants were performed with 25 μL of 0.5 mg/mL protein and 5 μL of 5x SYPRO^™^ Orange (Thermo Scientific) in triplicate using a CFX96^™^ real-time PCR detection system (Bio-Rad). Fluorescence data was fitted using the CFX manager 2.1 (Bio-Rad).

### WCR anatomy and immunohistochemistry

Neonate WCR larvae fed on a diet overlaid with 5.9 μg/cm^2^ Mpp75Aa1.1 were collected and dissected for insect gut morphology assessment at different time periods. To obtain paraffin sections, entire larvae were fixed in phosphate buffer saline (PBS), pH 6.9, containing 4% paraformaldehyde, overnight at 4°C, washed in PBS containing 0.5% (v/v) Triton X-100 (PBST) and dehydrated in a series of 20 min incubation steps with 50%, 75%, and 95% (v/v) ethanol (in ddH2O). Dehydrated samples were incubated in chloroform at room temperature for 4 h and subsequently in liquified paraffin at ~ 60°C overnight. Paraffin blocks were cut into 5 μm thick slices, deparaffinize, and rehydrated with xylene for 10 min, then with consecutive 5 min incubations in 100%, 96%, 70%, and 40% ethanol (in ddH2O) for 5 min, and in PBST for 10 min.

For immuno-blotting and staining, samples were blocked with 10% normal goat serum (NGS) (Thermo Fisher Scientific) in PBST for 40 min and rinsed 3 times with PBST. Primary polyclonal antibody against Mpp75Aa1.1 was added at 1:100 dilution in PBST at 4°C. After 3 consecutive washes of 5 min with PBST, sections were incubated with Alexa-Fluor-488 conjugated goat anti-rabbit IgG secondary antibody (Jackson Immuno-Research Laboratories, INC.) at room temperature for 4 h in the dark. Sections were subsequently washed in PBST three times (5 min each) and mounted in FluoroQuest Mounting Medium (AAT Bioquest) containing 4’,6’Diamidine-2’-phenylindole dihydrochloride (DAPI). Images were captured using a confocal microscope (Zeiss LSM 700).

## Results

### Mpp75Aa1.1 is structurally related to the ETX_MTX2 family of β-PFPs

**Fig 1:**
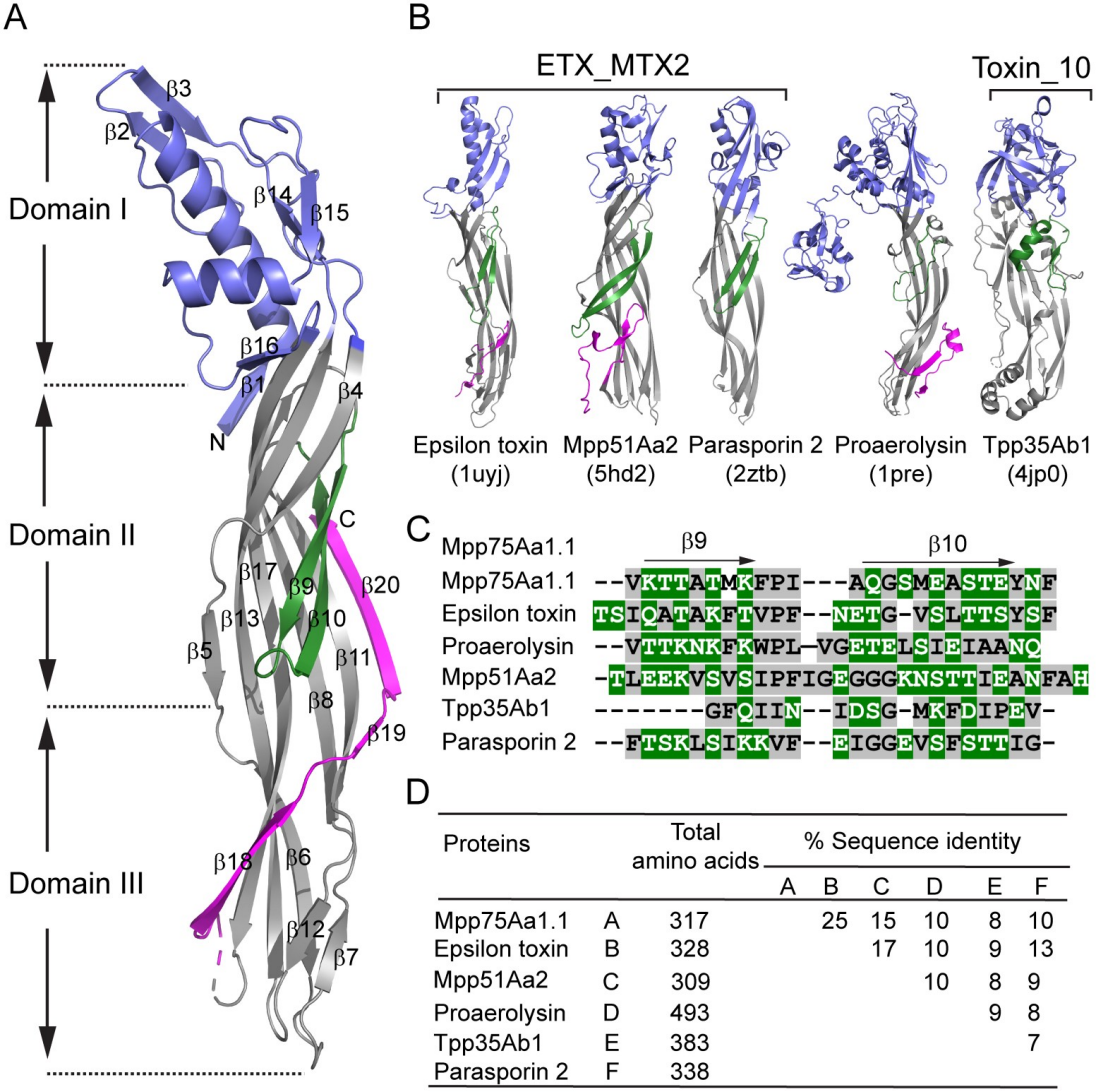
Structural and evolutionary properties of *Brevibacillus laterosporus* Mpp75Aa1.1, an ETX_MTX2 β-pore-forming protein. (A) Cartoon representation of the Mpp75Aa1.1 crystal structure. Domain I, the transmembrane pore-forming β-hairpin, and the carboxyl-terminal peptide, are colored in slate, green, and magenta, respectively. (B) Structural comparison of related β-PFPs Mpp51Aa2, Parasporin 2, Epsilon toxin, Proaerolysin, and Tpp35Ab1. All structural cartoons were rendered using PyMOL^™^2.0.2–Copyright. (C) Multiple sequence alignment (CLUSTAL W) of the transmembrane β-pore forming hairpin from Mpp75Aa1.1 and selected β-PFPs. Alternate hydrophobic (grey) and hydrophilic (green) amino acid residues are shown. (D) Pairwise amino acid sequence alignments of Mpp75Aa1.1 and selected β-PFPs. Alignments were obtained using CLCBio^™^ version 7.6.4. Primary amino acid sequences used are from Mpp75Aa1.1 (ASY04853.1), Epsilon toxin (AAA23235.1), Mpp51Aa2 (ADK94873.1), Parasporin 2 (BAC79010.1), Proaerolysin (WP_098980947.1), and Tpp35Ab1 (AAG41672.1).

The crystal structure of Mpp75Aa1.1 was solved at 1.94 Å resolution (S1 Table) using the SAD heavy atom phasing method from a crystal briefly soaked in samarium acetate. The protein crystallized in tetragonal crystal lattice of space group *P4*_*3*_*22* with one molecule in the asymmetric unit. The Mpp75Aa1.1 structure (Fig 1A) revealed an overall elongated shape with approximate dimensions of 112 Å x 26 Å x 22 Å with a high prevalence of β-strands. The molecule is composed of three structural domains (Fig 1A). Domain I, corresponding to the head region of the molecule, displays a non-contiguous fold encompassing four alpha-helices (α1-α4) and six short β-strands (β1-β3 and β14-β16) (Fig 1A). Additionally, domain I contains clusters of solvent-accessible aromatic residues. Domain II, at the center of Mpp75Aa1.1, is characterized by a set of anti-parallel β-strands running longitudinal across two-thirds of the Mpp75Aa1.1 structure, and a two-stranded anti-parallel β-hairpin (β9 and β10) which amino acid constituents are amphipathic, with alternating hydrophobic and hydrophilic residues (Fig 1A and 1C). Domain III, localized at the tail-end of Mpp75Aa1.1, contains a carboxyl terminal peptide (CTP), β-strands β18, β19, and β20 (β20 corresponds to an introduced histidine tag) (Fig 1A). An unstructured flexible loop delineates the N-terminal boundary of the CTP. Domains II-III adopts a topology representing the conserved structural core of the aerolysin-like proteins [8]. While the amino acid sequence analysis using InterPro [38] reveals that Mpp75Aa1.1 belongs to the ETX_MTX2 sub-family of the aerolysin-like β-PFPs, its three-dimensional architecture also confirms similarities as well as subtle but significant differences when compared to the crystal structure of other notable proteins in this protein family (Fig 1).

Proteins from the ETX_MTX2 family utilize a common pore-forming mechanism [13]. Structural alignments between Mpp75Aa1.1 and other well-characterized β-PFPs such as Mpp51Aa2, Parasporin-2, Epsilon toxin, Pro-aerolysin, and the WCR active Toxin_10 protein, Tpp35Ab1 (Fig 1B) were performed using the Secondary Structure Matching (SSM) superpose utility in Coot [39]. The root-mean-square deviation (rmsd) of the α-carbons revealed the following structural similarity between these proteins and Mpp75Aa1.1: Epsilon toxin (C_α_ rmsd = 2.4 Å) and Mpp51Aa2 (C_α_ rmsd = 4.1 Å), Parasporin-2 (C_α_ rmsd = 5.0 Å), Pro-aerolysin (C_α_ rmsd = 3.6 Å), and Tpp35Ab1(C_α_ rmsd = 4.5 Å) (S2 Table). In addition to the structural comparison across this panel of beta-pore forming protein representatives, primary amino acid sequence comparison reveals lower than 25% sequence identity (Fig 1D), which is largely based on homology in domains 2–3.

### Effects of solvent-exposed amino acids in Mpp75Aa1.1 domain I on insecticidal activity

**Fig 2:**
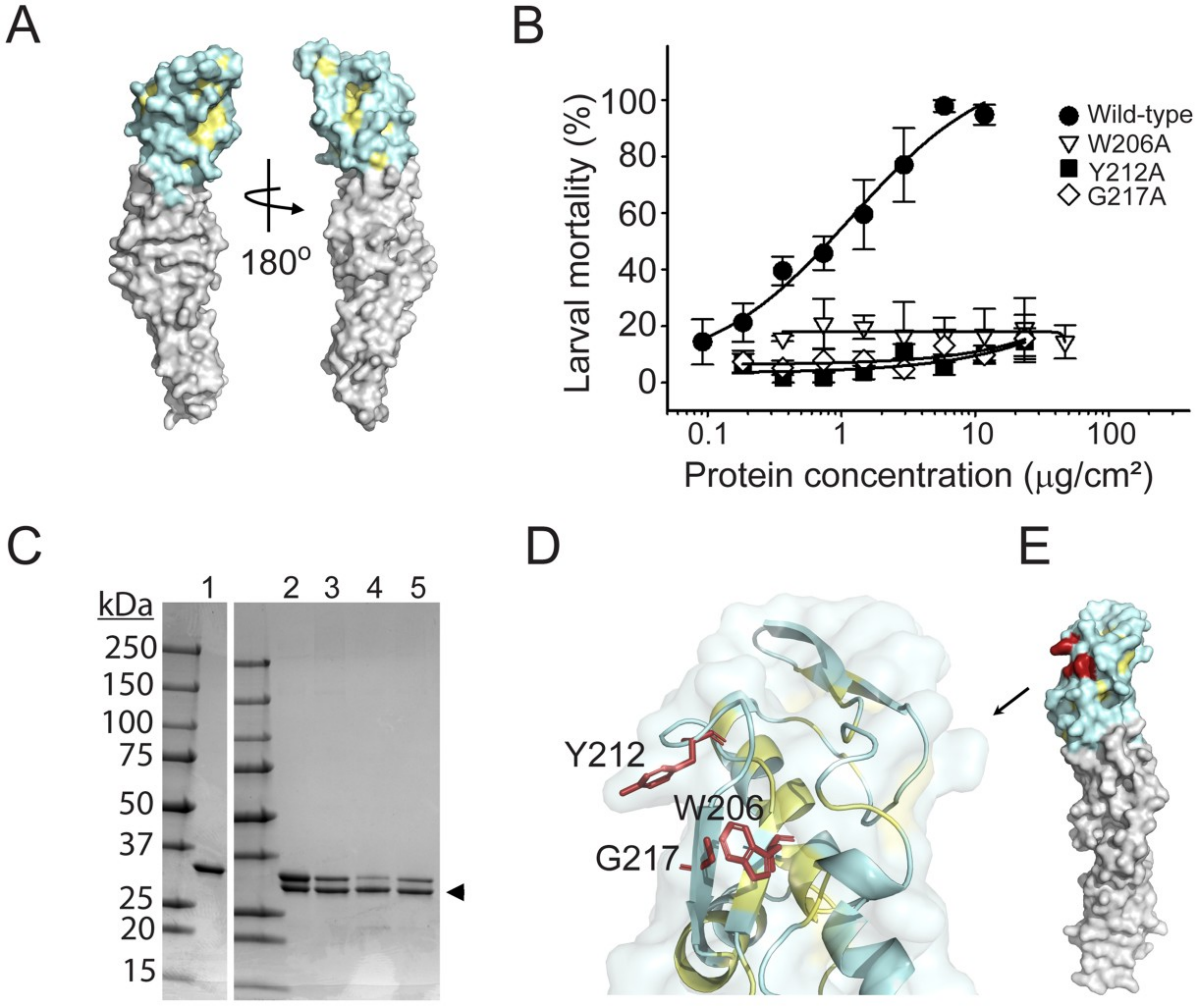
Domain I of Mpp75Aa1.1 comprises specific amino acids essential for insecticidal activity. (A) Surface representation of alanine-scanned residues in domain I. All domain I solvent-exposed amino acids substituted with alanine are colored in pale cyan. Partially buried amino acids are in pale yellow. (B) Concentration-dependent insecticidal activity of selected alanine variants against WCR larvae. Purified wild-type Mpp75Aa1.1 and variants W206A, Y212A, and G217A were used to treat WCR larvae in a diet overlay bioassay. All selected variants had significant reduction of insecticidal activity at concentrations as high as 23.5 μg/cm^2^. Data points represent mean mortality with standard error. (C) Proteolytic stability profile between Mpp75Aa1.1 and derived alanine variants. Wild-type (lane 1), processed wild-type (lane 2), and variants W206A (lane 3), Y212A (lane 4), and G217A (lane 5) are shown. The processed form of the proteins is indicated with the black arrowhead. (D) Key WCR-active residues of Mpp75Aa1.1 domain I are shown in red sticks. (E) Surface representation of the same residues. All structural renditions were from PyMOL^™^2.0.2.

A comprehensive alanine scanning substitution of all solvent-exposed amino acids in domain I (S4 Table), spanning amino acids 2 to 65 and 200 to 246, was performed to investigate whether solvent-exposed amino acids in the putative Mpp75Aa1.1 binding domain I are involved in WCR insecticidal activity (Fig 2A). Whole *E*. *coli* cells expressing recombinant wild-type Mpp75Aa1.1 and its derived alanine variants (71) were fed to WCR on artificial diet. Loss of insecticidal activity was initially detected with cells expressing alanine variants R11A, D34A, Y64A, K65A, W206A, Y212A, and G217A. Of these variants, R11A, D34A, Y64A, and K65A were further ruled out of this study due to either poor expression or high polydispersity; hence, whether R11A, D34A, Y64A, and K65A are integral to WCR toxicity remains unknown. Recombinant proteins W206A, Y212A, and G217A were then expressed and purified as described above. Dose-response assessment of insecticidal activity against WCR using purified alanine variants at concentration as high as 23.5 μg/cm^2^ revealed that W206A, Y212A, and G217A had a significant reduction of insecticidal activity (Fig 2B). Additional proteolysis and thermal-stability analyses were conducted to ascertain the structural integrity of the alanine variants. All three variants, W206A, Y212A, and G217A, exhibited a similar trypsin digestion profile (Fig 2C) and melting temperature (*T*_m_) as wild-type Mpp75Aa1.1. (S7 Table). Therefore, these alanine substitutions did not alter the tertiary structure of Mpp75Aa1.1, and reduced bioactivity is not caused by alteration of the tertiary structure, rather a change in Mpp75Aa1.1’s domain 1 function.

### Mpp75Aa1.1 has unique structural features in the WCR-specificity conferring domains I and III

**Fig 3:**
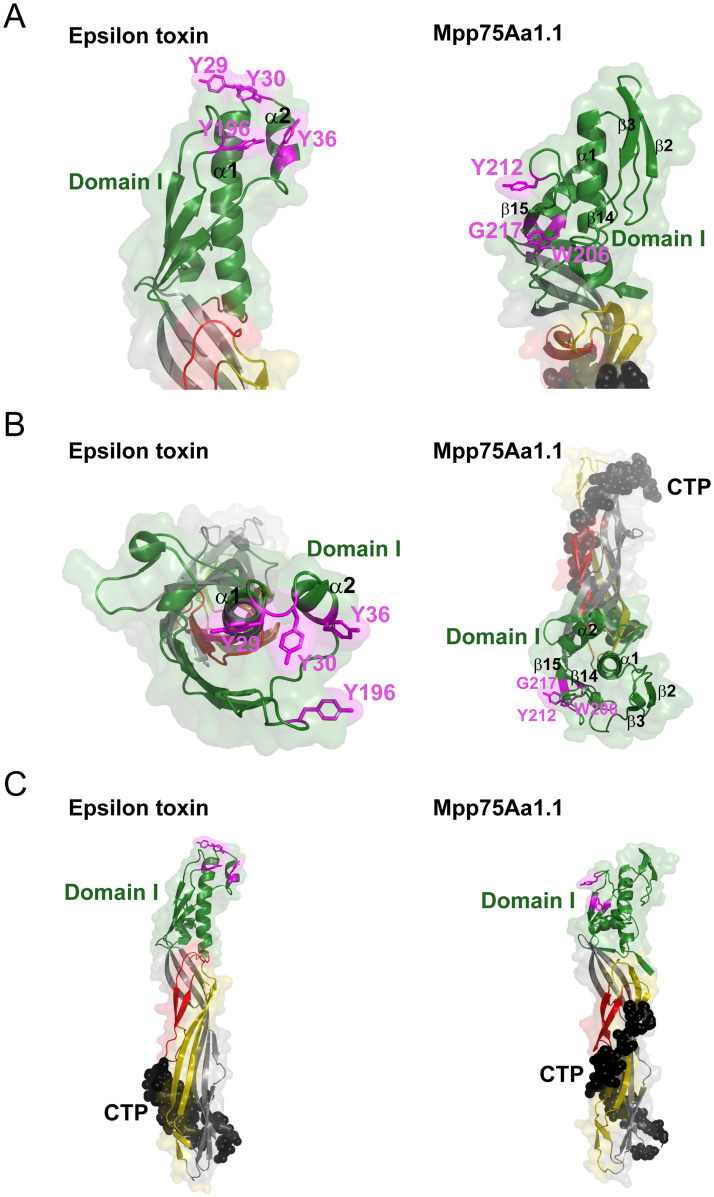
Structural comparison of Mpp75Aa1.1 and Epsilon toxin (ETX). (A) Head-to-head comparison between Mpp75Aa1.1 and ETX receptor binding domain, highlighting key residues. Domain I residues are shown in green and in cartoon representation; key residues are highlighted in magenta using sticks representation. (B) Top-down view of Mpp75Aa1.1 and ETX along the longitudinal axis of helix α1, highlighting potential key receptor binding epitopes, and additional domains in the full-length proteins. Domain I residues are shown in green cartoon representation. Domains II and III residues are indicated in red, yellow, and grey colors in cartoon representation, which correspond to strands that comprise the membrane-inserted pore (red), pore extension (yellow) and outer barrel (grey) portion in the membrane-inserted β-hairpin of these proteins; the color assignment was based on the available sequence and structural homology of Mpp75Aa1.1. Key receptor binding residues are highlighted in magenta using sticks representation. (C) The ETX and Mpp75Aa1.1 structure highlighting the C-terminal peptide residues in black spheres including the seven-residue C-terminal his-tag. Domain I residues are shown in green and in cartoon representation. Domain II and III residues are indicated with red, yellow and grey colors in cartoon representation, corresponding to strands that comprise the membrane-inserted pore (red), pore extension (yellow) and outer barrel (grey) portion in the membrane-inserted pore state of these proteins; the color assignment was based on the available sequence and structural homology of Mpp75Aa1.1. Key receptor binding residues are highlighted in magenta using sticks representation.

We further analyzed how the likely binding determinants of Mpp75Aa1.1 are related to the receptor binding domain of the structurally similar Epsilon toxin (ETX). For ETX, three of the four key binding residues in its receptor binding region are located immediately after helix α1 in an unstructured loop as well as in helix α2 (Fig 3A) [16]. While a long helix, α1, is also present in Mpp75Aa1.1, a longer domain 1 segment follows and folds into two anti-parallel β-strands (β2 and β3, Fig 3A). Furthermore, the Mpp75Aa1.1 structure also revealed that WCR-specificity is likely conferred by three domain I residues in the β14 (W206), β15 (G217) strands as well as in the connecting unstructured loop (Y212), which are located at a different part of domain I (Fig 3A and 3B).

Another unique structural feature of Mpp75Aa1.1 is its bent shape (Fig 3B). When the full-length structures are rendered along the axis of helix α1 in domain I, the ETX domains appear to be stacked, whereas Mpp75Aa1.1 domains are curved away from this axis. Lastly, our structure also revealed unique structural features at the C-terminus. We observed a more extended C-terminal peptide that encircles parts of domain II of Mpp75Aa1.1 via hydrogen bonded interactions between β17 and β18 as well as β20 and β11 (excluding the his-tag residues), whereas ETX has a C-terminal peptide that folds back on itself, thereby reducing the extent of the interactions with the adjacent domain II β-strands (Fig 3C).

### Effects of amino acid substitutions on the β-hairpin function

**Fig 4:**
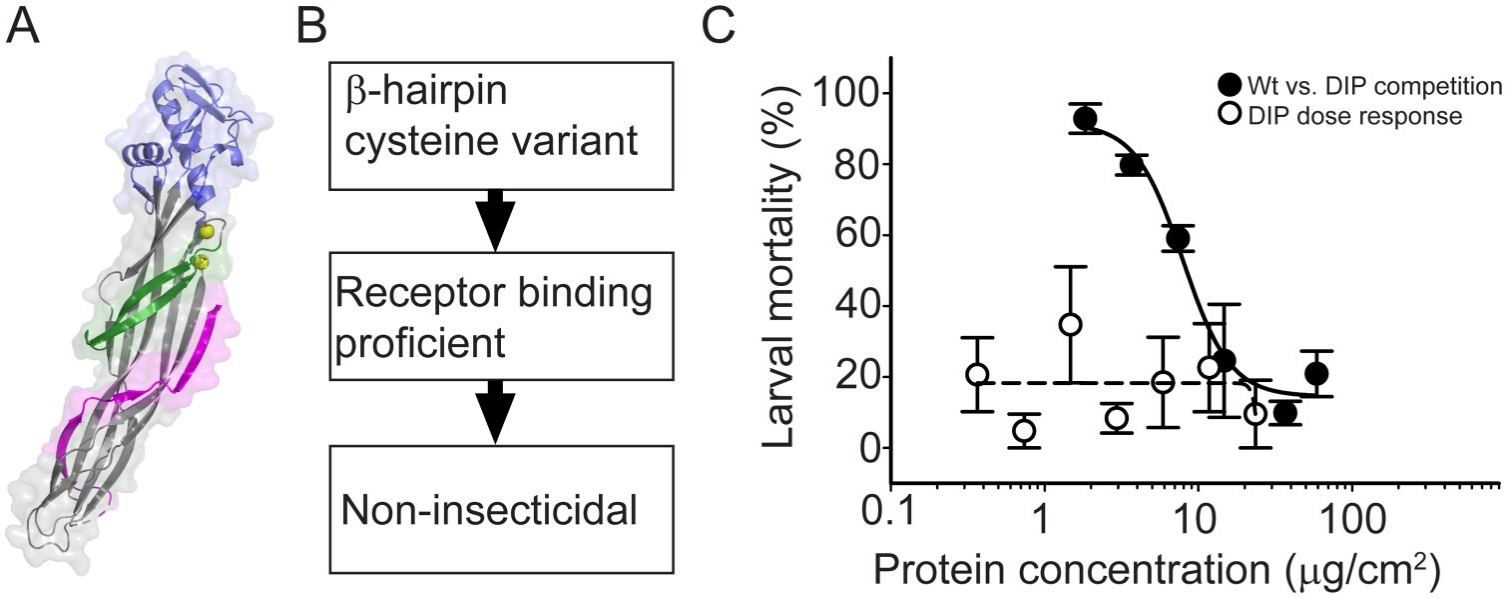
Competition bioassay with wild-type and disabled insecticidal protein (DIP) variant Mpp75Aa1.1_K125C_N153C. (A) Cartoon representation (PyMOL^™^2.0.2) of DIP variant. Double-cysteine substitutions K125C_N153C are represented by yellow spheres. (B) Description of DIP variant functional properties. (C) Mass action *in vivo* competition. Wild-type Mpp75Aa1.1 (7.35 μg/cm^2^) was mixed with increasing concentrations (from 1.83 to 58.8 μg/cm^2^) of the DIP-variant to measure WCR larval mortality. DIP-variant loss of insecticidal activity, likely caused by disabled pore formation, did not affect binding, as shown by effective competition. Data points represent mean mortality (± S.E.).

To determine whether the Mpp75Aa1.1 β-hairpin could possibly be involved in forming pores, consistent with other β-PFPs, we generated double cysteine substitutions near the β-hairpin to suppress its structural re-arrangement. Among several double-cysteine variants screened for loss of insecticidal activity, variant Mpp75Aa1.1_K125C_N153C (Fig 4A) was selected as a Mpp75Aa1.1 disabled insecticidal protein (DIP) variant. Competition bioassay analysis between Mpp75Aa1.1 and its DIP variant (Fig 4C) demonstrated that the non-cross-linked DIP variant was effective in competing away the insecticidal activity of the wild-type Mpp75Aa1.1 protein. Because Mpp75Aa1.1_K125C_N153C domain I was not altered, we deduced that it was unable to form pores but could compete with the wild-type protein using its intact binding domain as has been previously reported [18].

### Binding of Mpp75Aa1.1 to WCR microvilli promotes damage of the insect midgut epithelium

**Fig 5:**
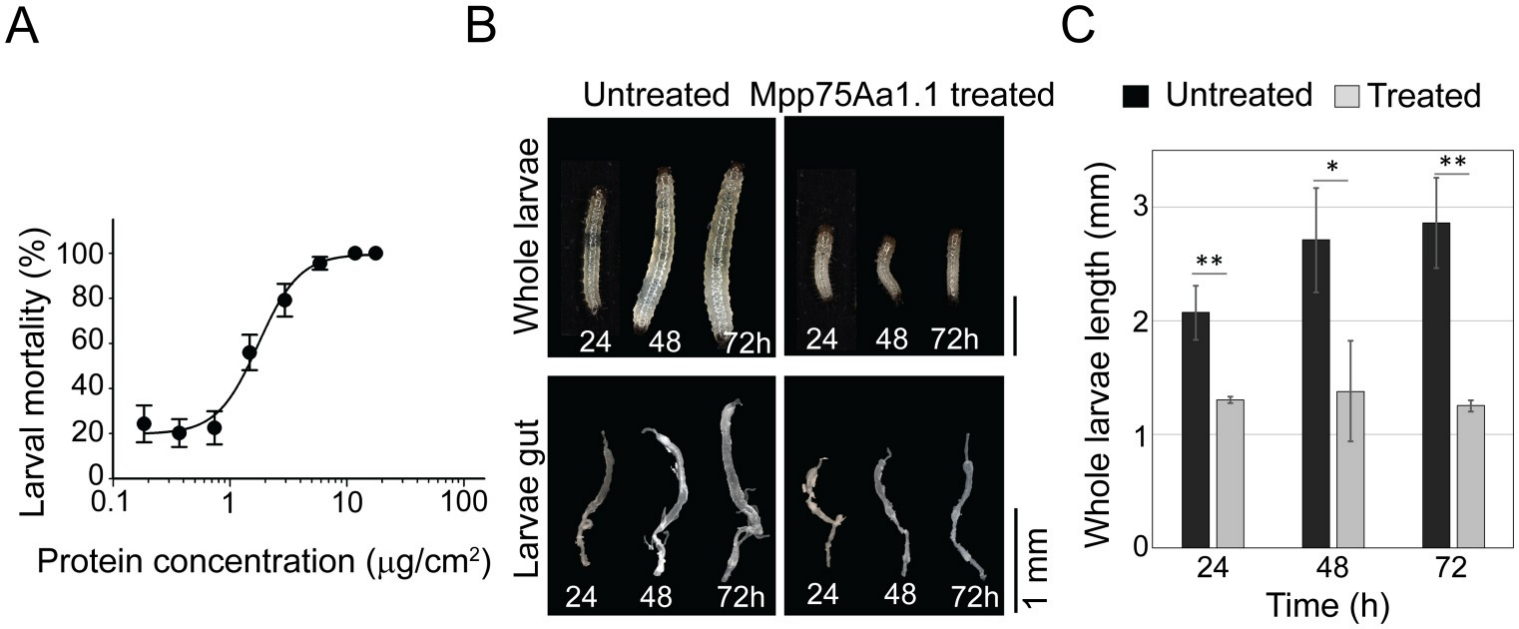
Effects of Mpp75Aa1.1 on WCR larvae development and dissected gut morphology. (A) Insecticidal activity of Mpp75Aa1.1 measured by increasing protein dose. Data points represent mean mortality (± S.E.), not corrected for buffer control mortality (4.75% ± 4.76). (B) Whole insect growth inhibition and isolated gut morphology assessments after 24 h, 48 h, and 72 h exposure to Mpp75Aa1.1. Significant insect stunting observed as early as 24 h post-exposure. (C) Bar graph representation of growth inhibition. Asterisks indicate statistical significance at p = 0.05 (*) and p = 0.01 (**) using the student t-test (n = 5).

**Fig 6:**
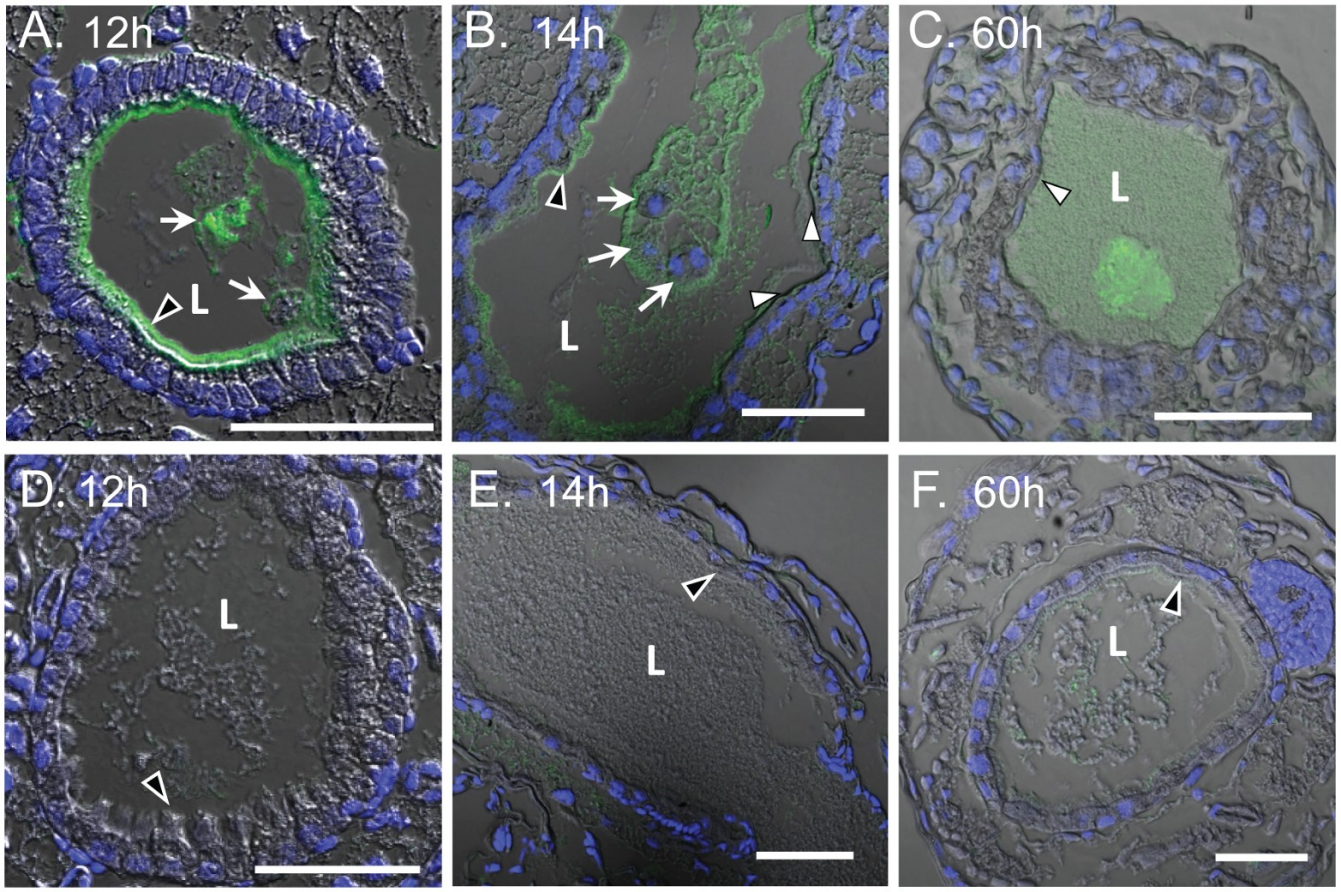
Immunohistochemistry assessment of WCR midgut morphology after feeding with Mpp75Aa1.1. (A) Cross-section of WCR larvae midgut immuno-detected with anti-Mpp75Aa1.1 antibody (green) and stained with the nucleus staining dye, DAPI (blue), after 12 h exposure to Mpp75Aa1.1. Mpp75Aa1.1 binding to intact midgut microvilli is designated by a black arrowhead. White arrows show sloughed off epithelial cellular debris in the lumen (L). (B) Longitudinal section after 14 h exposure to Mpp75Aa1.1. White arrowhead indicates complete to near-complete loss of the apical microvilli layer. (C) Cross-section after 60 h exposure to Mpp75Aa1.1. Complete loss of the apical microvilli is shown by white arrowhead. (D), (E), and (F) are corresponding untreated controls where black arrowheads designate intact apical microvilli layer. Scale bars are all 50 μm.

Morphological assessment of WCR larvae treated with Mpp75Aa1.1 exhibited stunted growth as early as 24 h post-exposure (Fig 5B and 5C). Subsequent analysis of dissected guts from 24 h to 72 h-intoxicated larvae displayed reduced size (Fig 5B) and were uncharacteristically brittle. Immunohistochemistry analysis of Mpp75Aa1.1 pathology on WCR larvae demonstrated protein binding to the microvilli and the sloughing off of the apical microvilli layer in the insect gut lumen after 12 to 14 h exposure (Fig 6A and 6B). Longer exposure of 60 h led to severe sloughing off of the apical microvilli layer in most cross-sections analyzed (70% of the 39 larvae tested). While intense immuno-staining of the apical microvilli layer and luminal contents was observed after 12 h exposure, the untreated controls had no signal (Fig 6A, 6C and 6D).

### WCR-specific gut fluid is required for proteolytic processing at the C-terminus, and subsequent self-oligomerization of Mpp75Aa1.1

**Fig 7:**
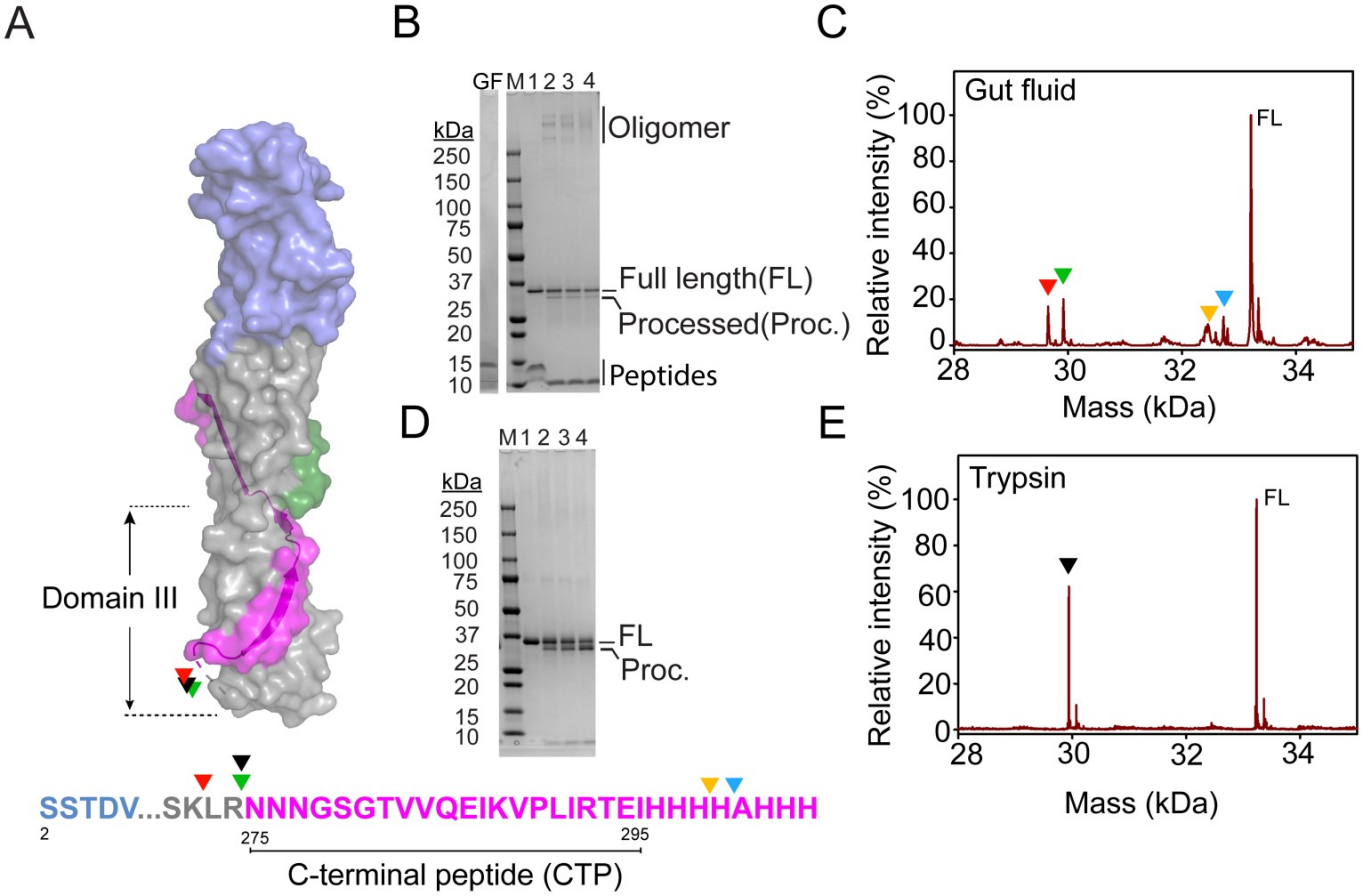
Protease processing of Mpp75Aa1.1 carboxyl-terminal peptide (CTP) yields a stable core with the propensity to form oligomers. (A) Surface rendition of Mpp75Aa1.1 domain III. The CTP is in magenta. Protease cleavage sites are colored arrowheads on the surface structure as well as on the primary amino acid sequence. (B) *In vitro* time course of Mpp75Aa1.1 proteolytic processing with WCR gut fluid at pH 6.0. Gut fluid (GF) only and oligomer formation are indicated. Lane 1 indicates unprocessed Mpp75Aa1.1; lane 2, lane 3, and lane 4 are processed Mpp75Aa1.1 at 10 min, 30 min, and 60 min, respectively. (C) Mass spectrometry profile of *in vitro* processed Mpp75Aa1.1 with WCR gut fluid. Cleaved polypeptide fragments are indicated by red (29.67 kDa), green (29.94 kDa), yellow (32.61 kDa), and cyan (32.75 kDa) arrowheads. (D) *In vitro* time course of Mpp75Aa1.1 proteolytic processing using trypsin. Lane 1 represents unprocessed Mpp75Aa1.1; lane 2, lane 3, and lane 4 are processed Mpp75Aa1.1 at 10 min, 15 min, and 30 min, respectively. (E) Mass spectrometry profile of *in vitro* processed Mpp75Aa1.1 with trypsin. Processed polypeptide is indicated by the black (29.94 kDa) arrowhead.

The susceptibility of Mpp75Aa1.1 to proteolytic processing is illustrated in Fig 7A, where both WCR gut fluid proteases and trypsin primarily target a flexible loop N-terminal to the CTP. To analyze the proteolytic processing of Mpp75Aa1.1, a time course for proteolysis was conducted with WCR gut fluid extract from 3^rd^ instar larvae at physiological pH 6.0 and room temperature. Within 10 min of incubation with gut fluid, Mpp75Aa1.1 was processed to a ~29 kDa protein (Fig 7B and 7C). Additionally, a portion of the cleaved Mpp75Aa1.1 formed SDS-resistant oligomers of approximately 250 kDa, 315 kDa, and 350 kDa (Fig 7B). Oligomer bands were further confirmed as Mpp75Aa1.1 (S6 Table) by in-gel peptide mapping using mass spectrometry [36,40].

Whereas E-64 inhibits both cysteine proteases and trypsin, PMSF is a serine protease inhibitor. When proteolytic activity of WCR gut fluid was blocked by both E-64 and PMSF, processing of Mpp75Aa1.1 was greatly reduced, and oligomers were not formed (Fig 7C). The inhibition study with both E-64 and PMSF indicates that the dominant protease that processes Mpp75Aa1.1 is likely a trypsin-like serine protease.

Amino-terminal sequencing of both the *in vitro*-processed and unprocessed forms of Mpp75Aa1.1 by Edman degradation [41] revealed that only the first methionine was removed at the amino-terminus of the protein, but significant proteolytic cleavage occurred at the carboxyl-terminus. The CTP cleavage sites were subsequently mapped by mass spectrometry analysis (Fig 7C), and they were identified as positions 274 (arginine) and 272 (lysine) under WCR gut fluid digestion (Fig 7C), and position 274 when trypsin was used (Fig 7E). Additionally, the trypsin processed Mpp75Aa1.1 retained similar insecticidal activity (S5 Table).

## Discussion

In the current study, we provide insights into the WCR activity of Mpp75Aa1.1 based on sequence, structure, and functional characterization. The structure of Mpp75Aa1.1, determined by X-ray crystallography, revealed a protein of three distinct domains and rich in β-strands arranged in a similar conformation as the conserved structural core of the β-PFPs from the ETX_MTX2 family [8,9,11,15]. The Mpp75Aa1.1 structure also contains an amphipathic β-hairpin in domain II similar to other structures within the ETX_MTX2 protein family that is used to form pores [42]. Structural superposition analysis of its α-carbons derived a structural resemblance to the domains II and III of epsilon toxin [43] and the insecticidal protein Mpp51Aa2.834_16 [44], both members of the ETX_MTX2 family of proteins. These proteins share very little primary amino acid sequence similarities with Mpp75Aa1.1, which like other ETX_MTX2 proteins, has a structurally diversified binding domain I (Fig 1B) that confers cell-type specificity, highlighting the distinct function of different domains [17]. Our Mpp75Aa1.1 structure also uncovered another unique structural feature in its bent shape shown on Fig 3B. While the significance of this structural feature is not known, its importance can be appreciated given the general mechanistic steps involved in the pore-forming MOA of these proteins: domain I residues (green) provide access to receptor and thus proximity to the membrane, domain 2/3 residues (grey) assist in self-oligomerization provided activation and CTP cleavage are complete, and then strands indicated with red and yellow rearrange and insert into the membrane. While the general mechanistic steps are the same for these proteins, Mpp75Aa1.1’s unique bent shape may have importance in stabilizing the protoxin prior to the specific protease activation or eliminate steric hindrance upon interaction with the protease or the receptor. Additionally, our head-to-head comparison between the monomeric structure of Mpp75Aa1.1 and the notable mammalian toxin, ETX, already revealed significant structural and sequence differences at the HAVCR1 receptor binding region of ETX [45] and the C-terminal end (Fig 3A). Lack of structural homology to the ETX receptor binding epitope suggests that the WCR-active Mpp75Aa1.1 would not have preference for that receptor. Indeed, the Mpp75Aa1.1 structure shows that WCR-specificity conferring residues are located in the β14 (W206), β15 (G217) strands as well as in the connecting unstructured loop (Y212), which are all part of domain I, but comprise a different epitope location (Fig 3A). Overall, Mpp75Aa1.1 represents the first in its family of ETX_MTX2 proteins to exhibit insecticidal activity against WCR, and our structural and functional characterization results uncovered structural features in domains I and III that contribute to the observed WCR-specificity.

The Mpp75Aa1.1 CTP is susceptible to protease processing. The acidic pH of the WCR midgut [46], or contact with the cell membrane, likely promotes release of the CTP upon proteolysis. It is possible that significant conformational changes take place to drive insertion of the β-hairpin into the cell membrane to form pores [14,47,48]. The role of the Mpp75Aa1.1 CTP is likely to prevent premature oligomerization or pore-formation in solution (or in the absence of the target cell) as has been observed for the CTPs from aerolysin and epsilon toxin [49]. More recently, the *Lygus*-active Mpp51Aa2.834_16 head-to-tail dimer protein was shown to dissociate into its monomeric form when processed by proteases at the carboxyl-terminus. This step mediated by the CTP of Mpp51Aa2.834_16 promotes its activation, oligomerization, and an increased binding affinity to a putative receptor [18]. A related mechanism is also found with the 130–145 kDa 3D-Cry insecticidal proteins (Cry1, Cry4, and Cry9). These proteins have a large carboxyl-terminal protoxin which, when solubilized and exposed to the insect midgut environment, is proteolytically processed to a stable active core [50–52], representing an activation mechanism for this structural class of insecticidal proteins. Overall, the role of Mpp75Aa1.1 CTP in protein activation and subsequent oligomer formation step parallels other ETX_MTX2 β-PFPs and 3D-Cry insecticidal proteins. Furthermore, our results showing that Mpp75Aa1.1’s *in vitro* protease processing can take place at a broad pH regime (depending on the source of the trypsin-like serine protease), but the subsequent self-oligomerization step is only favored by the WCR midgut-specific neutral to slightly acidic pH would suggest that this step may also contribute to WCR specificity of this insecticidal protein. This may likely be connected to the unique domain III structural feature of Mpp75Aa1.1 in that it has an extended C-terminal peptide that encircles parts of domain II via hydrogen bonded interactions between β17 and β18 as well as β20 and β11 (excluding the his-tag residues). This extensive interaction between strands of the CTP and domain 2/3 strands in Mpp75Aa1.1 suggests that oligomerization (via grey strands, Fig 3C) and subsequent pore-formation (yellow/red strands, Fig 3C) may not only require a protease cleavage, but also the complete separation of the CTP peptide from Mpp75Aa1.1 in a pH-dependent manner. This structural insight may have significance in the overall specificity of Mpp75Aa1.1 as removal of the CTP could require specific midgut milieu in addition to a specific protease.

Putative pore-forming proteins of distinct structures target different cell-types by recognizing different membrane associated receptors [15]. Receptor recognition, mediated by the diversified binding domains of the β-PFPs, can be one of the steps that confers insecticidal specificity. Previous investigations [16,53] have uncovered, within the structurally diverse domain I of epsilon toxin, amino acid residues with low sequence conservation as critical modulators of cell surface receptor recognition. Furthermore, structural and functional analyses have provided supportive evidence for cognate receptor recognition by ETX_MTX2-like proteins [16,17,53] as well as other insecticidal proteins [54]. Stemming from these studies, an alanine scanning mutagenesis of all surface-exposed amino acids in the putative Mpp75Aa1.1 binding domain I was performed to identify critical residues with potential altered binding properties and insecticidal activity toward WCR. Thermal stability and proteolytic processing compared to wild-type Mpp75Aa1.1 were used to rule out potential conformational changes associated with the alanine substitutions. We found that solvent-exposed amino acids W206, Y212, and G217, within the putative binding domain I of Mpp75Aa1.1, were integral to WCR susceptibility. Given that these residues are on the surface of the putative receptor binding domain of Mpp75Aa1.1 and that they are in close spatial proximity, we hypothesize that they comprise the toxin/receptor binding interface. Thus, reduced insecticidal activity would correlate with loss of binding. Perhaps more importantly, the spatial clustering of the Mpp75Aa1.1 binding determinants identified in the current investigation are unique from binding determinants found in Epsilon toxin [53], one of the well-characterized ETX_MTX2 protein, suggesting a distinct WCR midgut protein recognition motif for Mpp75Aa1.1.

The overall high prevalence of aromatic amino acids in the binding interfaces of ETX_MTX2 proteins, often defined by tryptophan and tyrosine residues, provides a unique configuration where aromatic side chains can stack with sugar rings [55,56] for specific receptor recognition. Therefore, it is conceivable that W206, Y212, and G217 are capable of stacking and interacting with carbohydrate elements on a putative glycoprotein receptor. Moreover, the WCR-active Tpp35Ab1, a β-PFP constituent of the binary toxin from the Toxin-10 family, exhibits a β-trefoil carbohydrate-binding motif within its domain I, suggesting possible glycan interaction [57,58]. Similar β-trefoil modules have been described for the mosquitocidal binary β-PFPs, BinAB [59], where the BinB component was reported to recognize a membrane associated α-glucosidase glycoprotein [60], further emphasizing the role of glycoprotein receptors in the molecular mechanism of action of insecticidal β-PFPs. Future research will investigate the functional receptor for Mpp75Aa1.1 in WCR, although many functional bacterial insecticidal protein receptors are identified only after resistance to the target insect has occurred [61].

Pore formation and midgut tissue damage are the final mechanistic steps proposed for bacterial insecticidal protein activity in most reported cases [50]. In the larvae of the southern corn rootworm, (SCR), *Diabrotica undecimpunctata*, ingested proteins travel the alimentary canal, to exert their mode of action in the midgut [62]. Ryerse *et al*. [62] described the gut anatomy of SCR as comprising a nutrient permeable peritrophic membrane lamellae formation stacked onto the microvilli along the length of the midgut epithelium. In the current study, immuno-histological analysis was performed on WCR larvae fed Mpp75Aa1.1. The histology of the WCR midgut indicates binding of Mpp75Aa1.1 to the apical microvilli of the midgut epithelium. Furthermore, significant sloughing off of cellular debris into the lumen was observed 12 h post-exposure, suggesting the midgut microvilli as a target site for Mpp75Aa1.1. The WCR midgut microvilli was also a target site for the Gpp34Ab1/Tpp35Ab1 binary proteins [63]. In contrast to Mpp75Aa1.1 intoxication, large vacuoles and blebbing off of large vesicles were observed with the use of Gpp34Ab1/Tpp35Ab1. In a subsequent investigation, ingestion of Cry3Aa1 and Cry6Aa1 by WCR promoted similar midgut tissue damage as that of Gpp34Ab1/Tpp35Ab1 [64]. Together, these studies show that putative pore-forming proteins go through similar mechanistic steps, which lead to presumed pore-formation and subsequent midgut damage. While the observed midgut damage can be different for different putative pore-forming proteins, for Mpp75Aa1.1, it coincides with stunted growth and insect mortality as reported for other bacterial insecticidal proteins [18,63,64].

In conclusion, this work has established that Mpp75Aa1.1, a new ETX_MTX2 protein, confers WCR activity and shares a similar mode of action to other Mpp, Tpp, and Cry proteins. Furthermore, this study also demonstrates that the combination of a few key protein attributes, namely protein stability in target pest milieu, proteolytic activation, and unique amino acid residues in the putative binding domain I, could confer different biological specificities for sequence-diverse representatives of the ETX_MTX2 family of proteins.

## Supporting information

S1 Fig Mpp75Aa1.1 binds to WCR BBM. (A) Solution binding of trypsin-treated and iodoacetamide fluorescein (IAF)-labeled double cysteine variant Mpp75Aa1.1_K125C_N153C (Mpp75Aa1.1_C-IAF_Tt). Mpp75Aa1.1_C-IAF_Tt was competed with increasing challenge ratio of trypsin treated unlabeled Mpp75Aa1.1_C_Tt. (B) Bar graph illustration of panel B. Bars represent the mean band-intensity of three experimental repeats with standard error. Mean values with the same letter are not statistically different (One Way ANOVA Student-Newman-Keuls’ test, α = 0.05).
(TIF)

S2 Fig WCR gut fluid processing of Mpp75Aa1.1 carboxyl-terminal peptide triggers oligomer formation, that is suppressed by the cysteine protease inhibitor (E-64) and the serine protease inhibitor (PMSF). (A) Time course *in vitro* processing of Mpp75Aa1.1 with WCR gut fluid. Wild-type (Wt) only and gut fluid (GF) only lanes are indicated. Black, white, and grey arrowheads respectively indicate oligomer bands 1, 2, and 3 identified as Mpp75Aa1.1 by in-gel peptide mapping using mass spectrometry. (B) Time course *in vitro* processing of Mpp75Aa1.1 with trypsin. Wild-type (Wt) only lanes are indicated. (C) Time course proteolytic processing and effects of protease inhibitors (E-64 and PMSF) on oligomer formation, (D) Gel image of gut fluid only lane used as negative control in Fig 7. Lanes 1,2,3, and 4 are not relevant to the current studies.
(TIF)

S3 Fig Full gels and blots represented in Fig 7 and S1 Fig. (A) Proteolytic stability profile between Mpp75Aa1.1 and derived alanine variants. Wild-type (lane 1), processed wild-type (lane 2), and variants W206A (lane 3), Y212A (lane 4), and G217A (lane 5) are shown. (B) Solution binding of trypsin-treated and iodoacetamide fluorescein (IAF)-labeled double cysteine variant Mpp75Aa1.1_K125C_N153C (Mpp75Aa1.1_C-IAF_Tt). Mpp75Aa1.1_C-IAF_Tt was competed with increasing challenge ratio (1:1 and 1:15) of trypsin treated unlabeled Mpp75Aa1.1_C_Tt. (C) Un-stained gel imaged in panel (B). Marker lane is shown.
(TIF)

S1 Table Structure solution and refinement parameters. (DOCX)

S2 Table Pairwise structural alignments of full length Mpp75Aa1.1 and selected homologs using alpha-carbon (C_α_). ^1^Underlined rmsd determined with less than 40% of the C_α_ from the reference structure are deemed unreliable. The Mpp75Aa1.1 reference structure has 295 residues; 118 residues correspond to 40%. Number of aligned C_α_ is reported in parenthesis.
(DOCX)

S3 Table Pairwise structural alignments of Mpp75Aa1.1 domains II-III and that of selected homologs using alpha-carbon (C_α_). ^1^Underlined rmsd determined with less than 40% of the C_α_ from the reference structure are deemed unreliable. Number of aligned C_α_ is reported in parenthesis.
(DOCX)

S4 Table Surface-exposed Mpp75Aa1.1 domain I amino acids substituted to alanine. (DOCX)

S5 Table Susceptibility of WCR larvae to insecticidal proteins. ^a^ Trypsin treated (Tt) Mpp75Aa1.1. ^b^ Assay buffer of 25 mM Na carbonate pH 10.5 and 25 mM NaCl. ^c^ Means followed by an asterisk are significantly different from buffer control treatment at *P*-value ≤ 0.038.
(DOCX)

S6 Table In-gel peptide mapping using mass spectrometry identifies oligomer as Mpp75Aa1.1 after 10 min proteolytic processing by gut fluid. (DOCX)

S7 Table Analysis of thermal stability. (DOCX)

S1 File Structure 1000256098 val-report-full P1. (PDF)
