# Supplementary figures and images for: Structural and functional characterization of Mpp75Aa1.1, a putative beta-pore forming protein from Brevibacillus laterosporus active against the western corn rootworm
Source: PLoS One. 2021 Oct 11;16(10):e0258052. doi: 10.1371/journal.pone.0258052 (PMC8504720; doi:10.1371/journal.pone.0258052)

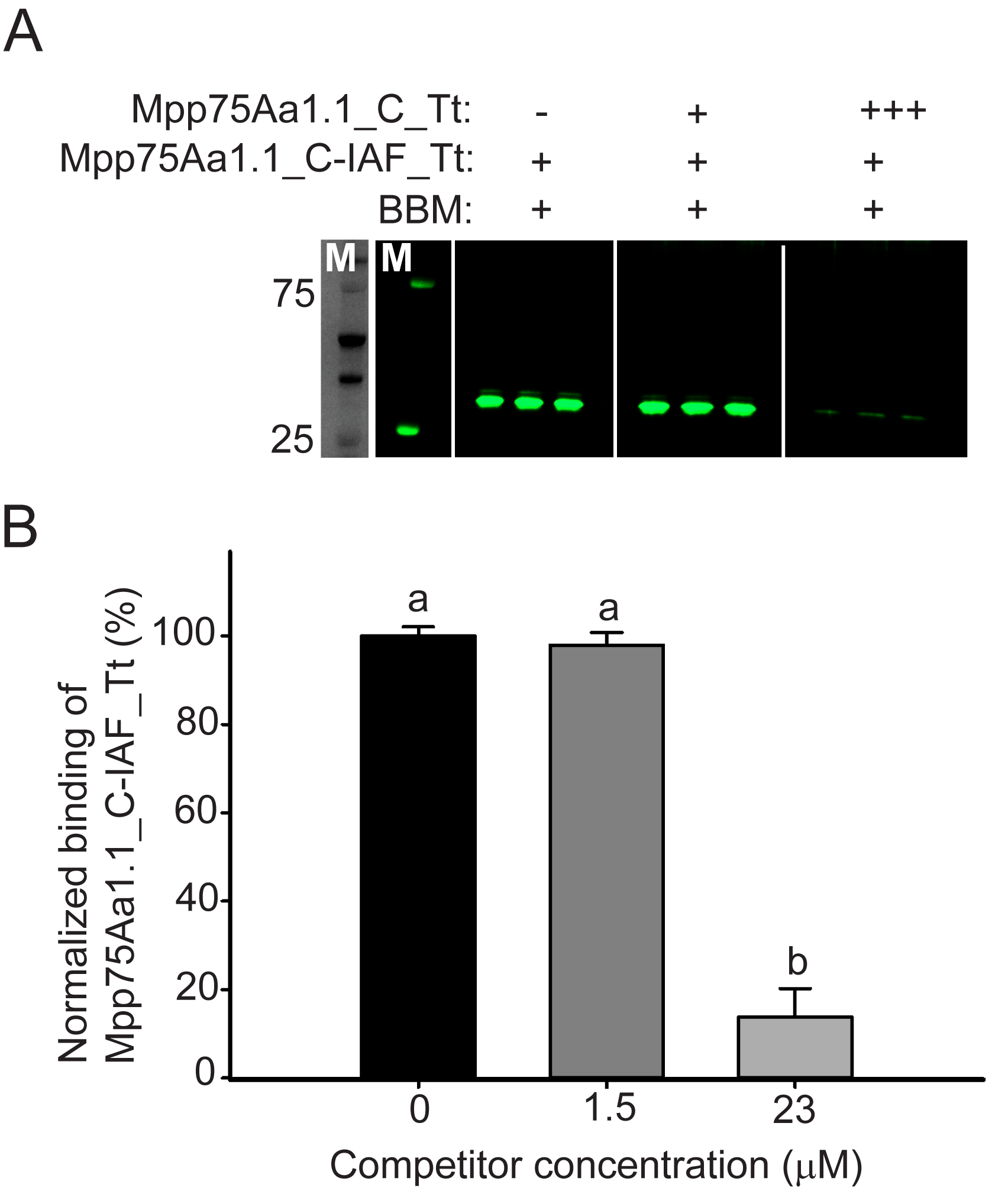

Supplement: S1 Fig — (A) Solution binding of trypsin-treated and iodoacetamide fluorescein (IAF)-labeled double cysteine variant Mpp75Aa1.1_K125C_N153C (Mpp75Aa1.1_C-IAF_Tt). Mpp75Aa1.1_C-IAF_Tt was competed with increasing challenge ratio of trypsin treated unlabeled Mpp75Aa1.1_C_Tt. (B) Bar graph illustration of panel B. Bars represent the mean band-intensity of three experimental repeats with standard error. Mean values with the same letter are not statistically different (One Way ANOVA Student-Newman-Keuls’ test, α = 0.05). (TIF) [file pone.0258052.s001.tif]

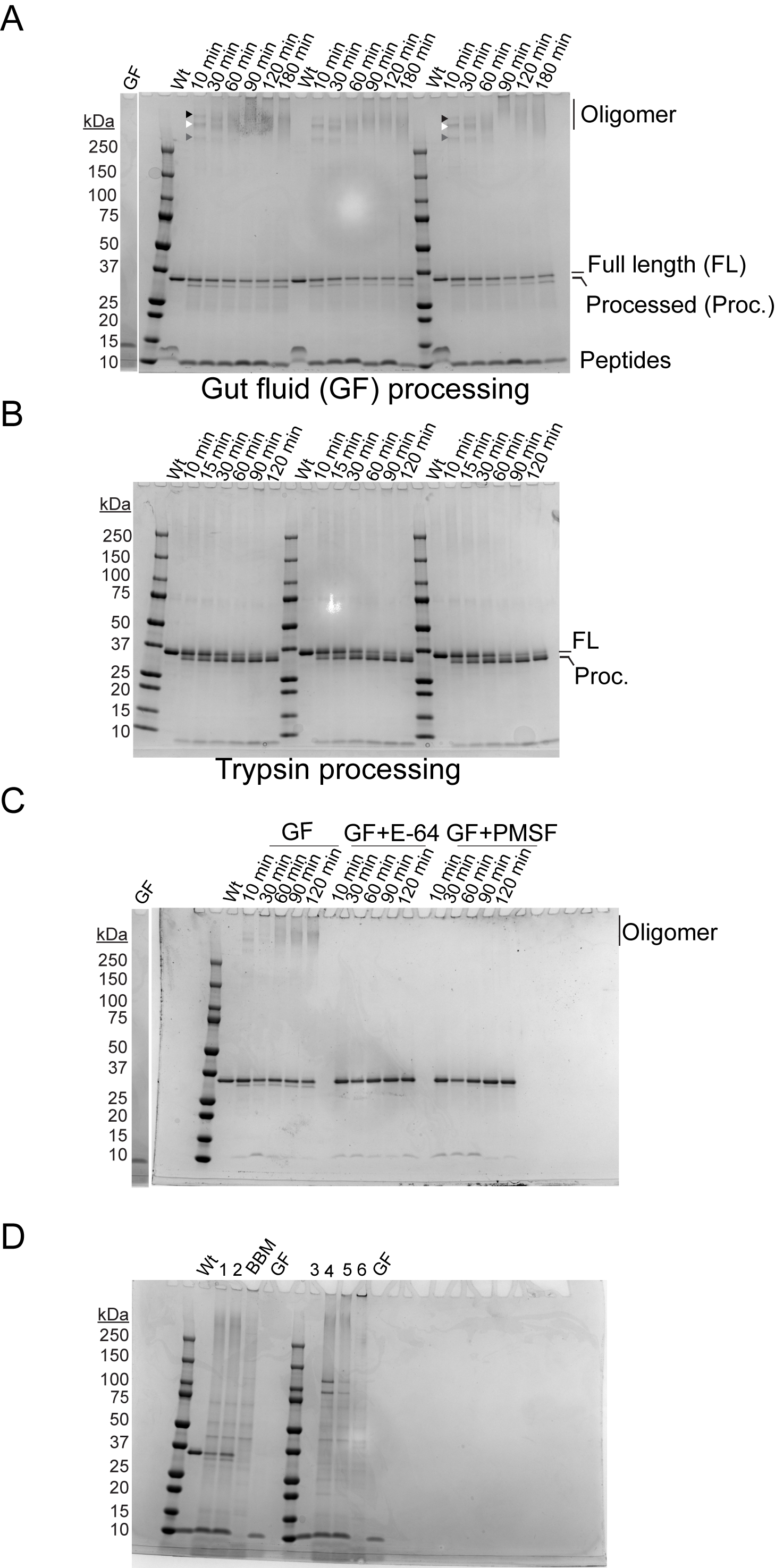

Supplement: S2 Fig — (A) Time course in vitro processing of Mpp75Aa1.1 with WCR gut fluid. Wild-type (Wt) only and gut fluid (GF) only lanes are indicated. Black, white, and grey arrowheads respectively indicate oligomer bands 1, 2, and 3 identified as Mpp75Aa1.1 by in-gel peptide mapping using mass spectrometry. (B) Time course in vitro processing of Mpp75Aa1.1 with trypsin. Wild-type (Wt) only lanes are indicated. (C) Time course proteolytic processing and effects of protease inhibitors (E-64 and PMSF) on oligomer formation, (D) Gel image of gut fluid only lane used as negative control in Fig 7. Lanes 1,2,3, and 4 are not relevant to the current studies. (TIF) [file pone.0258052.s002.tif]

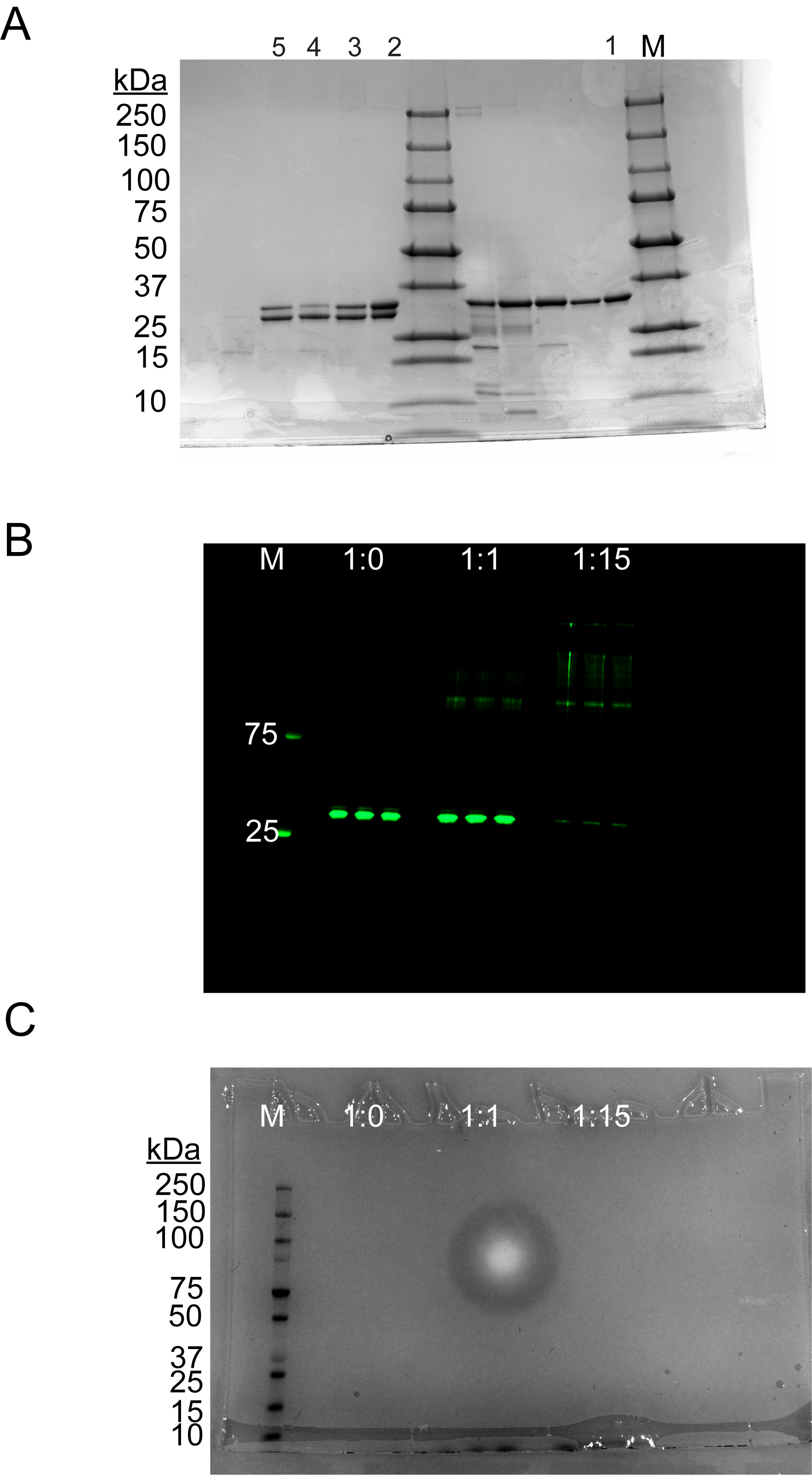

Supplement: S3 Fig — (A) Proteolytic stability profile between Mpp75Aa1.1 and derived alanine variants. Wild-type (lane 1), processed wild-type (lane 2), and variants W206A (lane 3), Y212A (lane 4), and G217A (lane 5) are shown. (B) Solution binding of trypsin-treated and iodoacetamide fluorescein (IAF)-labeled double cysteine variant Mpp75Aa1.1_K125C_N153C (Mpp75Aa1.1_C-IAF_Tt). Mpp75Aa1.1_C-IAF_Tt was competed with increasing challenge ratio (1:1 and 1:15) of trypsin treated unlabeled Mpp75Aa1.1_C_Tt. (C) Un-stained gel imaged in panel (B). Marker lane is shown. (TIF) [file pone.0258052.s003.tif]
