# Supplementary material for: Structural and functional characterization of Mpp75Aa1.1, a putative beta-pore forming protein from Brevibacillus laterosporus active against the western corn rootworm
Source: PLoS One. 2021 Oct 11;16(10):e0258052. doi: 10.1371/journal.pone.0258052 (PMC8504720; doi:10.1371/journal.pone.0258052)
Supplement: S1 Table — (DOCX) [file pone.0258052.s004.docx]

| Space group | *P4_3_22* |
| --- | --- |
| Unit cell lengths (Å) | a = b=69.8, c = 241.2 |
| Unit cell angles (°) | 90, 90, 90 |
| Resolution (Å) | 1.94-39.69 |
| No. of reflections | 584745 |
| Redundancy | 13.2 |
| <I/σ(I)> | 42.9 |
| Completeness (%) | 98.4 |
| R_merge-linear_ | 0.076 |
| R_merge-square_ | 0.077 |
| R_work_ | 21.9% |
| R_free_ | 24.7% |
| Protein residues/atoms | 295/2548 |
| Rmsd., bonds (A˚) | 0.007 |
| Rmsd., angles (°) | 0.830 |
| Ramachandran preferred (%) | 97.9% |
| Ramachandran allowed (%) | 2.1% |
