# Supplementary material for: Structural and functional characterization of Mpp75Aa1.1, a putative beta-pore forming protein from Brevibacillus laterosporus active against the western corn rootworm
Source: PLoS One. 2021 Oct 11;16(10):e0258052. doi: 10.1371/journal.pone.0258052 (PMC8504720; doi:10.1371/journal.pone.0258052)
Supplement: S2 Table — 1Underlined rmsd determined with less than 40% of the Cα from the reference structure are deemed unreliable. The Mpp75Aa1.1 reference structure has 295 residues; 118 residues correspond to 40%. Number of aligned Cα is reported in parenthesis. (DOCX) [file pone.0258052.s005.docx]

|  |  | Root mean square deviations^1^ (rmsd) on C_α_, in Å | | | | | |
| --- | --- | --- | --- | --- | --- | --- | --- |
|  |  | A | B | C | D | E | F |
| Mpp75Aa1.1 | A |  | 2.4 (188) | 4.1 (205) | 5.0 (174) | 3.6 (78) | 4.5 (79) |
| Epsilon toxin | B |  |  | 3.9 (201) | 3.9 (178) | 5.4 (166) | 4.3 (72) |
| Mpp51Aa2 | C |  |  |  | 5.1 (190) | 5.5 (170) | 5.9 (48) |
| Parasporin 2 | D |  |  |  |  | 4.6 (123) | 5.3 (77) |
| Proaerolysin | E |  |  |  |  |  | 7.7 (44) |
| Tpp35Ab1 | F |  |  |  |  |  |  |

Close

Distant
