# Supplementary material for: Structural and functional characterization of Mpp75Aa1.1, a putative beta-pore forming protein from Brevibacillus laterosporus active against the western corn rootworm
Source: PLoS One. 2021 Oct 11;16(10):e0258052. doi: 10.1371/journal.pone.0258052 (PMC8504720; doi:10.1371/journal.pone.0258052)
Supplement: S3 Table — 1Underlined rmsd determined with less than 40% of the Cα from the reference structure are deemed unreliable. Number of aligned Cα is reported in parenthesis. (DOCX) [file pone.0258052.s006.docx]

|  | Root mean square deviations ^1^ (rmsd) on C_α_, in Å | | | | | | |
| --- | --- | --- | --- | --- | --- | --- | --- |
|  |  | A | B | C | D | E | F |
| Mpp75Aa1.1 | A |  | 2.0 (159) | 2.9 (147) | 5.9 (68) | 4.4 (136) | 3.1 (72) |
| Epsilon toxin | B |  |  | 4.3 (142) | 3.0 (120) | 4.5 (131) | 4.3 (105) |
| Mpp51Aa2 | C |  |  |  | 3.6 (131) | 4.6 (141) | 5.0 (61) |
| Parasporin 2 | D |  |  |  |  | 3.6 (58) | 2.9 (93) |
| Proaerolysin | E |  |  |  |  |  | 4.4 (136) |
| Tpp35Ab1 | F |  |  |  |  |  |  |

Close

Distant
