# Supplementary material for: Structural and functional characterization of Mpp75Aa1.1, a putative beta-pore forming protein from Brevibacillus laterosporus active against the western corn rootworm
Source: PLoS One. 2021 Oct 11;16(10):e0258052. doi: 10.1371/journal.pone.0258052 (PMC8504720; doi:10.1371/journal.pone.0258052)
Supplement: S4 Table — (DOCX) [file pone.0258052.s007.docx]

| Domain I surface-exposed amino acids substituted to alanine | | | |
| --- | --- | --- | --- |
| S2 | D34 | R59 | G217 |
| S3 | E35 | S62 | V218 |
| T4 | Q36 | Q63 | N219 |
| D5 | Q37 | Y64 | T225 |
| Q7 | F38 | K65 | K226 |
| E8 | Y40 | V66 | Q228 |
| R9 | P42 | N67 | Q229 |
| R11 | T43 | N200 | K230 |
| D12 | E44 | G202 | G231 |
| R15 | G45 | I204 | D234 |
| T21 | I46 | W206 | R236 |
| N23 | V47 | S209 | N237 |
| E24 | F48 | P210 | Q239 |
| W26 | T50 | G211 | P240 |
| N27 | P52 | Y212 | S241 |
| N29 | K53 | P213 | G242 |
| K31 | N54 | N214 | R243 |
| P32 | G57 | G215 | D244 |
| S33 | E58 | G216 | I246 |
