# Supplementary material for: Structural and functional characterization of Mpp75Aa1.1, a putative beta-pore forming protein from Brevibacillus laterosporus active against the western corn rootworm
Source: PLoS One. 2021 Oct 11;16(10):e0258052. doi: 10.1371/journal.pone.0258052 (PMC8504720; doi:10.1371/journal.pone.0258052)
Supplement: S5 Table — a Trypsin treated (Tt) Mpp75Aa1.1. b Assay buffer of 25 mM Na carbonate pH 10.5 and 25 mM NaCl. c Means followed by an asterisk are significantly different from buffer control treatment at P-value ≤ 0.038. (DOCX) [file pone.0258052.s008.docx]

| Protein in this study | Concentration (µg/cm^2^) | Test set | Number of larvae per dose | Mean %  mortality (± SD) ^c^ |
| --- | --- | --- | --- | --- |
| Mpp75Aa1.1 | 1.5 | 1 | 24 | 43.45 ± 6.27* |
|  | 2.9 | 1 | 24 | 68.81 ± 7.84* |
|  | 5.9 | 1 | 24 | 76.19 ± 21.82* |
|  |  |  |  |  |
| Mpp75Aa1.1_Tt ^a^ | 1.5 | 1 | 24 | 37.50 ± 33.07 |
|  | 2.9 | 1 | 24 | 54.17 ± 19.09* |
|  | 5.9 | 1 | 24 | 84.72 ± 2.41* |
|  |  |  |  |  |
| Buffer Control ^b^ | 0.0 | 1 | 24 | 08.33 ± 14.43 |
