# Supplementary material for: Structural and functional characterization of Mpp75Aa1.1, a putative beta-pore forming protein from Brevibacillus laterosporus active against the western corn rootworm
Source: PLoS One. 2021 Oct 11;16(10):e0258052. doi: 10.1371/journal.pone.0258052 (PMC8504720; doi:10.1371/journal.pone.0258052)
Supplement: S6 Table — (DOCX) [file pone.0258052.s009.docx]

|  | Test 1 | | Test 2 | |
| --- | --- | --- | --- | --- |
| Mpp75Aa1.1 protein | Mpp75Aa1.1 Unique peptides identified | Mpp75Aa1.1 sequence coverage (%) | Mpp75Aa1.1 Unique peptides identified | Mpp75Aa1.1 sequence coverage (%) |
| Oligomer band 1 | 7 | 18.81 | 4 | 7.92 |
| Oligomer band 2 | 5 | 15.84 | 7 | 18.15 |
| Oligomer band 3 | 3 | 6.27 | 4 | 12.87 |
| Full length untreated | 22 | 58.09 | 22 | 60.07 |
