# Supplementary material for: Structural and functional characterization of Mpp75Aa1.1, a putative beta-pore forming protein from Brevibacillus laterosporus active against the western corn rootworm
Source: PLoS One. 2021 Oct 11;16(10):e0258052. doi: 10.1371/journal.pone.0258052 (PMC8504720; doi:10.1371/journal.pone.0258052)
Supplement: S7 Table — (DOCX) [file pone.0258052.s010.docx]

| Mpp75Aa1.1 and alanine variants | *T_m_* (± SE) |
| --- | --- |
| Wild-type | 38.00 ± 0.47 |
| W206A | 37.70 ± 0.24 |
| Y212A | 39.00 ± 0.41 |
| G217A | 37.80 ± 0.62 |
