# Supplementary material for: Structural and functional characterization of Mpp75Aa1.1, a putative beta-pore forming protein from Brevibacillus laterosporus active against the western corn rootworm
Source: PLoS One. 2021 Oct 11;16(10):e0258052. doi: 10.1371/journal.pone.0258052 (PMC8504720; doi:10.1371/journal.pone.0258052)
Supplement: S1 File — (PDF) [file pone.0258052.s011.pdf]

Full wwPDB X-ray Structure Validation Report (i)

Apr 29, 2021 – 10:49 AM EDT

PDB ID	:	7ML9
Title	:	The Mpp75Aa1.1 beta-pore-forming protein from Brevibacillus laterosporus
Deposited on	:	2021-04-27
Resolution	:	1.94 Å(reported)

This is a Full wwPDB X-ray Structure Validation Report.

This report is produced by the wwPDB biocuration pipeline after annotation of the structure.

We welcome your comments at *validation@mail.wwpdb.org*
A user guide is available at
https://www.wwpdb.org/validation/2017/XrayValidationReportHelp
with specific help available everywhere you see the (i) symbol.

CONFIDENTIAL VALIDATION REPORT

The following versions of software and data (see references (i)) were used in the production of this report:

MolProbity	:	4.02b-467
Mogul	:	1.8.5 (274361), CSD as541be (2020)
Xtriage (Phenix)	:	1.13
EDS	:	2.18
Percentile statistics	:	20191225.v01 (using entries in the PDB archive December 25th 2019)
Refmac	:	5.8.0158
CCP4	:	7.0.044 (Gargrove)
Ideal geometry (proteins)	:	Engh & Huber (2001)
Ideal geometry (DNA, RNA)	:	Parkinson et al. (1996)
Validation Pipeline (wwPDB-VP)	:	2.18

1 Overall quality at a glance (i)

The following experimental techniques were used to determine the structure:
X-RAY DIFFRACTION

The reported resolution of this entry is 1.94 Å.

Percentile scores (ranging between 0-100) for global validation metrics of the entry are shown in the following graphic. The table shows the number of entries on which the scores are based.

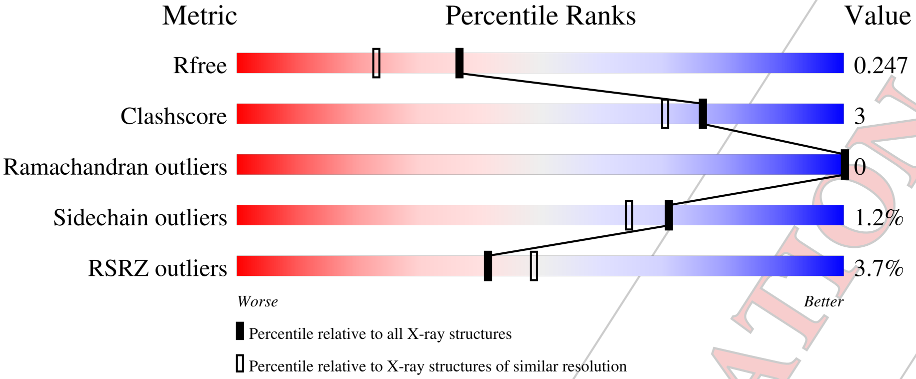

Metric	Whole archive (#Entries)	Similar resolution (#Entries, resolution range(Å))
R_{free}	130704	4310 (1.96-1.92)
Clashscore	141614	1023 (1.94-1.94)
Ramachandran outliers	138981	1007 (1.94-1.94)
Sidechain outliers	138945	1007 (1.94-1.94)
RSRZ outliers	127900	4250 (1.96-1.92)

The table below summarises the geometric issues observed across the polymeric chains and their fit to the electron density. The red, orange, yellow and green segments of the lower bar indicate the fraction of residues that contain outliers for >=3, 2, 1 and 0 types of geometric quality criteria respectively. A grey segment represents the fraction of residues that are not modelled. The numeric value for each fraction is indicated below the corresponding segment, with a dot representing fractions <=5% The upper red bar (where present) indicates the fraction of residues that have poor fit to the electron density. The numeric value is given above the bar.

Mol	Chain	Length	Quality of chain
1	A	325	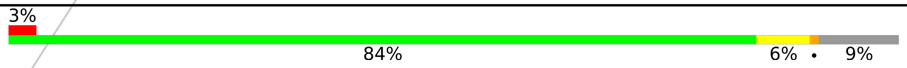

CONFIDENTIAL VALIDATION REPORT

2 Entry composition (i)

There are 6 unique types of molecules in this entry. The entry contains 2548 atoms, of which 0 are hydrogens and 0 are deuteriums.

In the tables below, the ZeroOcc column contains the number of atoms modelled with zero occupancy, the AltConf column contains the number of residues with at least one atom in alternate conformation and the Trace column contains the number of residues modelled with at most 2 atoms.

- Molecule 1 is a protein called Insecticidal protein.

Mol	Chain	Residues	Atoms					ZeroOcc	AltConf	Trace
			Total	C	N	O	S			
1	A	295	2321	1452	400	465	4	0	7	0

There are 8 discrepancies between the modelled and reference sequences:

Chain	Residue	Modelled	Actual	Comment	Reference
A	318	HIS	-	expression tag	UNP A0A249JQG5
A	319	HIS	-	expression tag	UNP A0A249JQG5
A	320	HIS	-	expression tag	UNP A0A249JQG5
A	321	HIS	-	expression tag	UNP A0A249JQG5
A	322	ALA	-	expression tag	UNP A0A249JQG5
A	323	HIS	-	expression tag	UNP A0A249JQG5
A	324	HIS	-	expression tag	UNP A0A249JQG5
A	325	HIS	-	expression tag	UNP A0A249JQG5

- Molecule 2 is SAMARIUM (III) ION (three-letter code: SM) (formula: Sm).

Mol	Chain	Residues	Atoms		ZeroOcc	AltConf
			Total	Sm		
2	A	2	2	2	0	0

- Molecule 3 is ACETATE ION (three-letter code: ACT) (formula: $C_2H_3O_2$).

CONFIDENTIAL VALIDATION REPORT

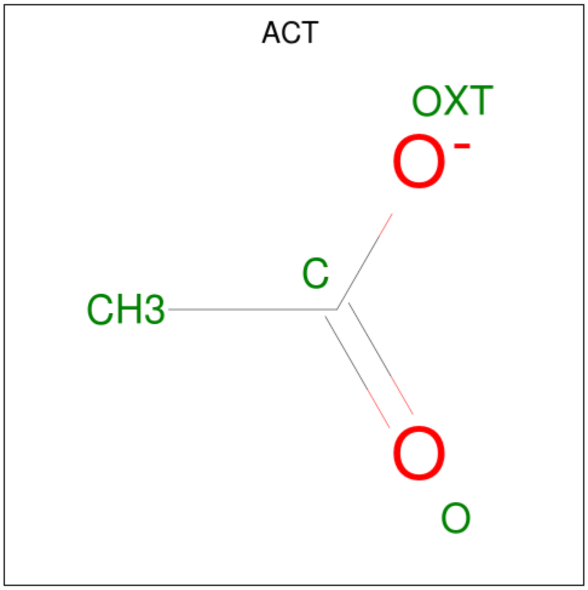

Mol	Chain	Residues	Atoms	ZeroOcc	AltConf
3	A	1	Total C O<br>4 2 2	0	0

- Molecule 4 is 1,2-ETHANEDIOL (three-letter code: EDO) (formula: $C_2H_6O_2$).

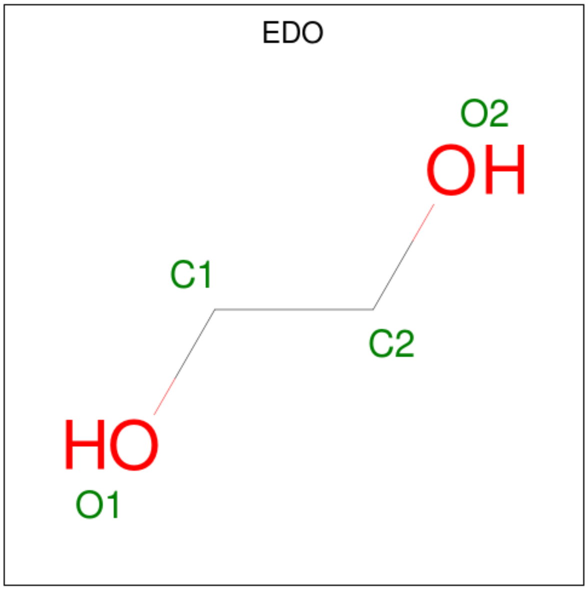

Mol	Chain	Residues	Atoms	ZeroOcc	AltConf
4	A	1	Total C O<br>4 2 2	0	0
4	A	1	Total C O<br>4 2 2	0	0
4	A	1	Total C O<br>4 2 2	0	0

Continued on next page...

CONFIDENTIAL VALIDATION REPORT

Continued from previous page...

Mol	Chain	Residues	Atoms	ZeroOcc	AltConf
4	A	1	Total C O<br>4 2 2	0	0

- Molecule 5 is SODIUM ION (three-letter code: NA) (formula: Na).

Mol	Chain	Residues	Atoms	ZeroOcc	AltConf
5	A	1	Total Na<br>1 1	0	0

- Molecule 6 is water.

Mol	Chain	Residues	Atoms	ZeroOcc	AltConf
6	A	204	Total O<br>204 204	0	0

CONFIDENTIAL VALIDATION REPORT

3 Residue-property plots (i)

These plots are drawn for all protein, RNA, DNA and oligosaccharide chains in the entry. The first graphic for a chain summarises the proportions of the various outlier classes displayed in the second graphic. The second graphic shows the sequence view annotated by issues in geometry and electron density. Residues are color-coded according to the number of geometric quality criteria for which they contain at least one outlier: green = 0, yellow = 1, orange = 2 and red = 3 or more. A red dot above a residue indicates a poor fit to the electron density (RSRZ > 2). Stretches of 2 or more consecutive residues without any outlier are shown as a green connector. Residues present in the sample, but not in the model, are shown in grey.

- Molecule 1: Insecticidal protein

Chain A:
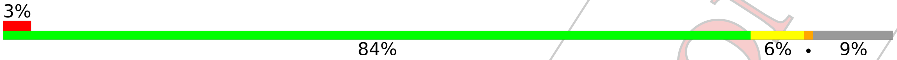

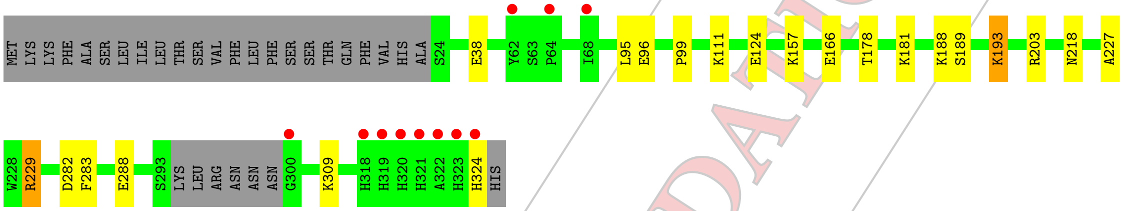

CONFIDENTIAL VALIDATION REPORT

4 Data and refinement statistics (i)

Property	Value	Source
Space group	P 43 2 2	Depositor
Cell constants<br>a, b, c, α, β, γ	69.79Å 69.79Å 241.24Å<br>90.00° 90.00° 90.00°	Depositor
Resolution (Å)	39.69 – 1.94<br>42.06 – 1.94	Depositor<br>EDS
% Data completeness<br>(in resolution range)	99.9 (39.69-1.94)<br>99.9 (42.06-1.94)	Depositor<br>EDS
R_{merge}	0.08	Depositor
R_{sym}	(Not available)	Depositor
$< I/\sigma(I) >$ [1]	3.29 (at 1.94Å)	Xtriage
Refinement program	PHENIX 1.17.1_3660	Depositor
R, R_{free}	0.219 , 0.247<br>0.219 , 0.247	Depositor<br>DCC
R_{free} test set	2278 reflections (5.04%)	wwPDB-VP
Wilson B-factor (Å^2)	26.4	Xtriage
Anisotropy	0.059	Xtriage
Bulk solvent k_{sol}(e/Å^3), B_{sol}(Å^2)	0.35 , 50.7	EDS
L-test for twinning[2]	$< \|L\| > = 0.48$, $< L^2 > = 0.31$	Xtriage
Estimated twinning fraction	No twinning to report.	Xtriage
F_o,F_c correlation	0.94	EDS
Total number of atoms	2548	wwPDB-VP
Average B, all atoms (Å^2)	31.0	wwPDB-VP

Xtriage's analysis on translational NCS is as follows: *The largest off-origin peak in the Patterson function is 4.10% of the height of the origin peak. No significant pseudotranslation is detected.*

[1] Intensities estimated from amplitudes.

[2] Theoretical values of $< |L| >$, $< L^2 >$ for acentric reflections are 0.5, 0.333 respectively for untwinned datasets, and 0.375, 0.2 for perfectly twinned datasets.

CONFIDENTIAL VALIDATION REPORT

5 Model quality (i)

5.1 Standard geometry (i)

Bond lengths and bond angles in the following residue types are not validated in this section: EDO, NA, ACT, SM

The Z score for a bond length (or angle) is the number of standard deviations the observed value is removed from the expected value. A bond length (or angle) with $|Z| > 5$ is considered an outlier worth inspection. RMSZ is the root-mean-square of all Z scores of the bond lengths (or angles).

Mol	Chain	Bond lengths		Bond angles	
		RMSZ	$\#|Z| > 5$	RMSZ	$\#|Z| > 5$
1	A	0.40	0/2392	0.58	0/3243

There are no bond length outliers.

There are no bond angle outliers.

There are no chirality outliers.

There are no planarity outliers.

5.2 Too-close contacts (i)

In the following table, the Non-H and H(model) columns list the number of non-hydrogen atoms and hydrogen atoms in the chain respectively. The H(added) column lists the number of hydrogen atoms added and optimized by MolProbity. The Clashes column lists the number of clashes within the asymmetric unit, whereas Symm-Clashes lists symmetry-related clashes.

Mol	Chain	Non-H	H(model)	H(added)	Clashes	Symm-Clashes
1	A	2321	0	2257	14	0
2	A	2	0	0	0	0
3	A	4	0	3	0	0
4	A	16	0	24	2	0
5	A	1	0	0	0	0
6	A	204	0	0	1	0
All	All	2548	0	2284	14	0

The all-atom clashscore is defined as the number of clashes found per 1000 atoms (including hydrogen atoms). The all-atom clashscore for this structure is 3.

All (14) close contacts within the same asymmetric unit are listed below, sorted by their clash magnitude.

CONFIDENTIAL VALIDATION REPORT

Atom-1	Atom-2	Interatomic distance (Å)	Clash overlap (Å)
1:A:227:ALA:O	1:A:229:ARG:NH1	2.24	0.70
1:A:203:ARG:NH1	4:A:408:EDO:O2	2.30	0.65
1:A:96:GLU:HG3	1:A:218:ASN:ND2	2.21	0.56
1:A:178:THR:OG1	1:A:324:HIS:HB3	2.08	0.54
1:A:124:GLU:HB3	1:A:193:LYS:HE3	1.91	0.53
1:A:188:LYS:NZ	6:A:507:HOH:O	2.42	0.53
1:A:96:GLU:HG3	1:A:218:ASN:HD21	1.74	0.52
1:A:157:LYS:NZ	1:A:166:GLU:HG2	2.25	0.51
1:A:282:ASP:OD2	1:A:309:LYS:HD3	2.12	0.49
1:A:189:SER:HB3	1:A:283:PHE:CD2	2.50	0.46
1:A:95:LEU:HD11	1:A:99:PRO:HG3	1.99	0.43
1:A:203:ARG:NH2	1:A:288:GLU:OE1	2.52	0.42
1:A:111:LYS:HE2	4:A:408:EDO:H22	2.02	0.41

There are no symmetry-related clashes.

5.3 Torsion angles (i)

5.3.1 Protein backbone (i)

In the following table, the Percentiles column shows the percent Ramachandran outliers of the chain as a percentile score with respect to all X-ray entries followed by that with respect to entries of similar resolution.

The Analysed column shows the number of residues for which the backbone conformation was analysed, and the total number of residues.

Mol	Chain	Analysed	Favoured	Allowed	Outliers	Percentiles	
1	A	298/325 (92%)	294 (99%)	4 (1%)	0	100	100

There are no Ramachandran outliers to report.

5.3.2 Protein sidechains (i)

In the following table, the Percentiles column shows the percent sidechain outliers of the chain as a percentile score with respect to all X-ray entries followed by that with respect to entries of similar resolution.

The Analysed column shows the number of residues for which the sidechain conformation was analysed, and the total number of residues.

CONFIDENTIAL VALIDATION REPORT

Mol	Chain	Analysed	Rotameric	Outliers	Percentiles	
1	A	257/278 (92%)	253 (98%)	4 (2%)	62	52

All (4) residues with a non-rotameric sidechain are listed below:

Mol	Chain	Res	Type
1	A	181[A]	LYS
1	A	181[B]	LYS
1	A	193	LYS
1	A	229	ARG

Sometimes sidechains can be flipped to improve hydrogen bonding and reduce clashes. All (1) such sidechains are listed below:

Mol	Chain	Res	Type
1	A	218	ASN

5.3.3 RNA (i)

There are no RNA molecules in this entry.

5.4 Non-standard residues in protein, DNA, RNA chains (i)

There are no non-standard protein/DNA/RNA residues in this entry.

5.5 Carbohydrates (i)

There are no monosaccharides in this entry.

5.6 Ligand geometry (i)

Of 8 ligands modelled in this entry, 3 are monoatomic - leaving 5 for Mogul analysis.

In the following table, the Counts columns list the number of bonds (or angles) for which Mogul statistics could be retrieved, the number of bonds (or angles) that are observed in the model and the number of bonds (or angles) that are defined in the Chemical Component Dictionary. The Link column lists molecule types, if any, to which the group is linked. The Z score for a bond length (or angle) is the number of standard deviations the observed value is removed from the expected value. A bond length (or angle) with $|Z| > 2$ is considered an outlier worth inspection. RMSZ is the root-mean-square of all Z scores of the bond lengths (or angles).

CONFIDENTIAL VALIDATION REPORT

Mol	Type	Chain	Res	Link	Bond lengths			Bond angles		
					Counts	RMSZ	#|Z| > 2	Counts	RMSZ	#|Z| > 2
4	EDO	A	404	-	3,3,3	0.40	0	2,2,2	0.61	0
3	ACT	A	403	2	1,3,3	8.69	1 (100%)	0,3,3	0.00	-
4	EDO	A	406	-	3,3,3	0.65	0	2,2,2	0.16	0
4	EDO	A	407	-	3,3,3	0.46	0	2,2,2	0.42	0
4	EDO	A	408	-	3,3,3	0.42	0	2,2,2	0.42	0

In the following table, the Chirals column lists the number of chiral outliers, the number of chiral centers analysed, the number of these observed in the model and the number defined in the Chemical Component Dictionary. Similar counts are reported in the Torsion and Rings columns. '-' means no outliers of that kind were identified.

Mol	Type	Chain	Res	Link	Chirals	Torsions	Rings
4	EDO	A	404	-	-	1/1/1/1	-
4	EDO	A	406	-	-	1/1/1/1	-
4	EDO	A	408	-	-	1/1/1/1	-
4	EDO	A	407	-	-	1/1/1/1	-

All (1) bond length outliers are listed below:

Mol	Chain	Res	Type	Atoms	Z	Observed(Å)	Ideal(Å)
3	A	403	ACT	CH3-C	8.69	1.59	1.48

There are no bond angle outliers.

There are no chirality outliers.

All (4) torsion outliers are listed below:

Mol	Chain	Res	Type	Atoms
4	A	406	EDO	O1-C1-C2-O2
4	A	408	EDO	O1-C1-C2-O2
4	A	404	EDO	O1-C1-C2-O2
4	A	407	EDO	O1-C1-C2-O2

There are no ring outliers.

1 monomer is involved in 2 short contacts:

Mol	Chain	Res	Type	Clashes	Symm-Clashes
4	A	408	EDO	2	0

CONFIDENTIAL VALIDATION REPORT

5.7 Other polymers (i)

There are no such residues in this entry.

5.8 Polymer linkage issues (i)

There are no chain breaks in this entry.

CONFIDENTIAL VALIDATION REPORT

6 Fit of model and data (i)

6.1 Protein, DNA and RNA chains (i)

In the following table, the column labelled '#RSRZ> 2' contains the number (and percentage) of RSRZ outliers, followed by percent RSRZ outliers for the chain as percentile scores relative to all X-ray entries and entries of similar resolution. The OWAB column contains the minimum, median, 95th percentile and maximum values of the occupancy-weighted average B-factor per residue. The column labelled 'Q< 0.9' lists the number of (and percentage) of residues with an average occupancy less than 0.9.

Mol	Chain	Analysed	<RSRZ>	#RSRZ>2			OWAB(Å^2)	Q<0.9
1	A	295/325 (90%)	0.22	11 (3%)	41	49	14, 27, 59, 78	0

All (11) RSRZ outliers are listed below:

Mol	Chain	Res	Type	RSRZ
1	A	324	HIS	6.2
1	A	323	HIS	4.9
1	A	62	TYR	4.8
1	A	300	GLY	4.1
1	A	321	HIS	3.2
1	A	319	HIS	2.8
1	A	64	PRO	2.6
1	A	68	ILE	2.5
1	A	322	ALA	2.4
1	A	318	HIS	2.3
1	A	320	HIS	2.3

6.2 Non-standard residues in protein, DNA, RNA chains (i)

There are no non-standard protein/DNA/RNA residues in this entry.

6.3 Carbohydrates (i)

There are no monosaccharides in this entry.

6.4 Ligands (i)

In the following table, the Atoms column lists the number of modelled atoms in the group and the number defined in the chemical component dictionary. The B-factors column lists the minimum,

CONFIDENTIAL VALIDATION REPORT

median, 95th percentile and maximum values of B factors of atoms in the group. The column labelled 'Q< 0.9' lists the number of atoms with occupancy less than 0.9.

Mol	Type	Chain	Res	Atoms	RSCC	RSR	B-factors(Å^2)	Q<0.9
3	ACT	A	403	4/4	0.72	0.20	35,45,47,53	0
4	EDO	A	406	4/4	0.84	0.21	32,37,40,41	0
4	EDO	A	407	4/4	0.84	0.17	32,36,37,51	0
4	EDO	A	404	4/4	0.88	0.18	27,29,33,40	0
4	EDO	A	408	4/4	0.89	0.24	47,53,54,61	0
2	SM	A	402	1/1	0.94	0.15	76,76,76,76	1
5	NA	A	405	1/1	0.95	0.14	28,28,28,28	0
2	SM	A	401	1/1	0.97	0.12	87,87,87,87	1

6.5 Other polymers ⓘ

There are no such residues in this entry.

CONFIDENTIAL VALIDATION REPORT
